# The Lactate‐Primed KAT8‒PCK2 Axis Exacerbates Hepatic Ferroptosis During Ischemia/Reperfusion Injury by Reprogramming OXSM‐Dependent Mitochondrial Fatty Acid Synthesis

**DOI:** 10.1002/advs.202414141

**Published:** 2025-01-24

**Authors:** Jingsheng Yuan, Mingyang Yang, Zhenru Wu, Jun Wu, Kejie Zheng, JiaGuo Wang, Qiwen Zeng, Menglin Chen, Tao Lv, Yujun Shi, Jiayin Yang, Jian Yang

**Affiliations:** ^1^ Liver Transplant Center Transplant Center West China Hospital Sichuan University Chengdu 610041 China; ^2^ Institute of Organ Transplantation Frontiers Science Center for Disease‐related Molecular Network West China Hospital of Sichuan University Chengdu 610041 China; ^3^ Key Laboratory of Transplant Engineering and Immunology NHC West China Hospital Sichuan University Chengdu 610041 China; ^4^ Department of Emergency and Critical Care Medicine West China School of Public Health West China Fourth Hospital Sichuan University Chengdu 610041 China; ^5^ Institute of Clinical Pathology Key Laboratory of Transplant Engineering and Immunology NHC West China Hospital Sichuan University Chengdu 610041 China

**Keywords:** ferroptosis, hepatic ischemia‒reperfusion injury, lactate, lactylation, metabolic reprogramming, mitochondrial fatty acid synthesis, phosphoenolpyruvate carboxykinase 2

## Abstract

Recipients often suffer from hyperlactatemia during liver transplantation (LT), but whether hyperlactatemia exacerbates hepatic ischemia‐reperfusion injury (IRI) after donor liver implantation remains unclear. Here, the role of hyperlactatemia in hepatic IRI is explored. In this work, hyperlactatemia is found to exacerbate ferroptosis during hepatic IRI. Lactate‐primed lysine acetyltransferase 8 (KAT8) is determined to directly lactylate mitochondrial phosphoenolpyruvate carboxykinase 2 (PCK2) at Lys100 and augments PCK2 kinase activity. By using gene‐edited mice, evidence indicating that PCK2 exacerbates hepatic ferroptosis during IRI is generated. Mechanistically, PCK2 lactylate at Lys100 acts as a critical inducer of ferroptosis during IRI by competitively inhibiting the Parkin‐mediated polyubiquitination of 3‐oxoacyl‐ACP synthase (OXSM), thereby leading to metabolic remodeling of mitochondrial fatty acid synthesis (mtFAS) and the potentiation of oxidative phosphorylation and the tricarboxylic acid cycle. More importantly, targeting PCK2 is demonstrated to markedly ameliorate hyperlactatemia‐mediated ferroptosis during hepatic IRI. Collectively, the findings support the use of therapeutics targeting PCK2 to suppress hepatic ferroptosis and IRI in patients with hyperlactatemia during LT.

## Graphical abstract

Recipients often suffer from hyperlactatemia during liver transplantation (LT). This study discovers that lactate‐primed KAT8 directly lactylates mitochondrial PCK2 at Lys100 (PCK2^K100lac^). PCK2^K100lac^ acts as a critical inducer of ferroptosis during ischemia‐reperfusion injury (IRI) by competitively inhibiting the Parkin‐mediated polyubiquitination of OXSM, thereby leading to metabolic remodeling of mitochondrial fatty acid synthesis. This process interprets hyperlactatemia‐mediated ferroptosis during hepatic IRI.

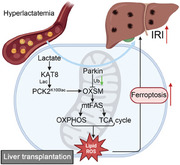

## 1 Introduction

Liver transplantation (LT) remains an indispensable treatment option for patients with end‐stage liver disease and liver malignancies.^[^ ^1^ ^]^ Despite the proposal of various novel perfusion strategies,^[^ ^2^ ^]^ hepatic ischemia‐reperfusion injury (IRI), a risk factor for primary graft dysfunction or nonfunction as well as acute and chronic rejection,^[^ ^3^ ^]^ remains inevitable. Identifying novel strategies to mitigate hepatic IRI would not only improve clinical outcomes but also enable the utilization of marginal liver grafts and thus expand the donor pool for life‐saving LT. Notably, due to increased anaerobic metabolism and reduced compensatory capacity, recipients often suffer from hyperlactatemia during LT surgery.^[^ ^4^ ^]^ Although clinical data indicate that hyperlactatemia is associated with primary graft dysfunction and mortality following LT,^[^ ^4^, ^5^ ^]^ whether hyperlactatemia exacerbates hepatic IRI after donor liver implantation remains unclear.

Recent studies have revealed that hyperlactatemia is strongly associated with disturbances in several metabolic pathways, such as the tricarboxylic acid (TCA) cycle and oxidative phosphorylation (OXPHOS),^[^ ^6^, ^7^ ^]^ both of which generate excess reactive oxygen species and may facilitate lipid peroxidation.^[^ ^8^ ^]^ Notably, lipid peroxidation is a direct trigger of ferroptosis. Ferroptosis, a newly discovered form of programmed cell death driven by iron‐dependent phospholipid peroxidation, has been shown to play an essential role in IRI to various organs.^[^ ^9^ ^]^ Several studies have demonstrated that the inhibition of ferroptosis can attenuate apoptosis, necrosis, and inflammatory infiltration during hepatic IRI.^[^ ^10^, ^11^ ^]^ Moreover, as the main organ for iron storage, the liver is more susceptible to ferroptosis than the heart and kidney are. Thus, we speculate that hyperlactatemia exacerbates ferroptosis during hepatic IRI. However, the role of ferroptosis in hyperlactatemia‐mediated IRI remains unclear, and revealing the potential regulatory mechanisms is crucial for developing effective therapies to mitigate hyperlactatemia‐mediated hepatic IRI.

Given the crucial involvement of mitochondria in various cellular processes, such as reactive oxygen species (ROS) production, energy metabolism, redox status, and iron metabolism, increasing evidence suggests a vital role for mitochondria in the regulation and execution of ferroptosis.^[^ ^12^ ^]^ Mitochondria are often thought of as predominantly catabolic organelles, breaking down carbon sources for use as fuel through fatty acid oxidation and the TCA cycle. In addition to these well‐known energy‐producing metabolic processes, a plethora of anabolic biochemical pathways, including those that generate nonessential amino acids, cholesterol, and steroid hormones, also occur in mitochondria. The mitochondrial fatty acid synthesis (mtFAS) pathway was first described in the yeasts *Saccharomyces cerevisiae* and *Neurospora crassa*, in which it was discovered to be required for the production of lipoic acid, a co‐factor required for several mitochondrial metabolic enzymes involved in OXPHOS and the TCA cycle, such as NADH:ubiquinone oxidoreductase subunit A6 (NDUFA6) and α‐ketoglutarate dehydrogenase (OGDH).^[^ ^13^, ^14^ ^]^ However, the potential role and related functional mechanisms of the mtFAS pathway in hepatic ferroptosis and IRI are largely unknown.

Here, we investigated the role of hyperlactatemia in ferroptosis during hepatic IRI. We found a positive correlation between hyperlactatemia and hepatic ferroptosis during LT surgery. We then determined that lactate‐primed lysine acetyltransferase 8 (KAT8) directly lactylates phosphoenolpyruvate carboxykinase 2 (PCK2) at Lys100 and increases PCK2 kinase activity. By using gene‐edited mice, we generated evidence indicating that PCK2 exacerbates hepatic ferroptosis during IRI. Mechanistically, lactylation of PCK2 at Lys100 suppresses Parkin‐mediated polyubiquitination of 3‐oxoacyl‐ACP synthase (OXSM), thereby leading to metabolic reprogramming to mtFAS, which in turn potentiates OXPHOS and TCA cycle activity. Importantly, we demonstrated that targeting PCK2 markedly ameliorates hyperlactatemia‐mediated ferroptosis during hepatic IRI. Collectively, our findings provide a novel PCK2‐targeted therapeutic strategy to suppress ferroptosis in patients with hyperlactatemia during hepatic IRI.

## 2 Results

### 2.1 Lactate Levels are Associated with Ferroptosis During Hepatic IRI

**Figure 1:**
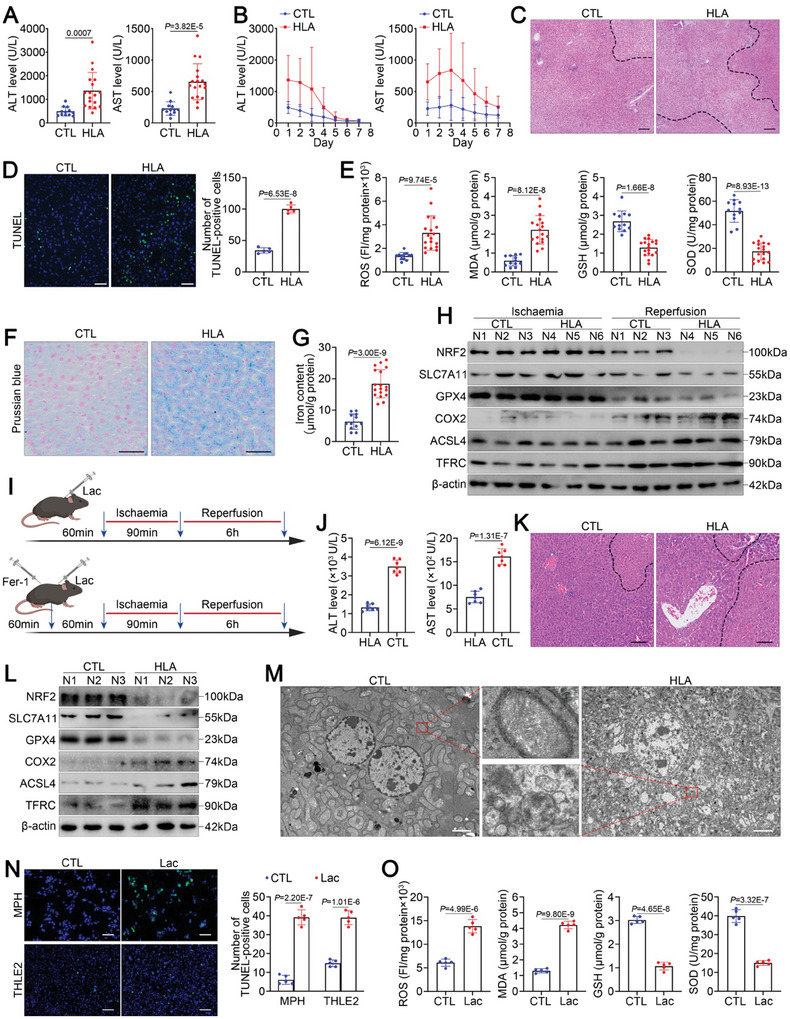
Hyperlactatemia aggravates ferroptosis during hepatic IRI. A) Serum AST and ALT levels in LT recipients in cohort 1 on POD1 (HLA group, *n* = 18; CTL group, *n* = 12). B) Curve of the serum ALT and AST levels in LT recipients in cohort 1 from POD1 to POD7 (HLA group, n = 18; CTL group, *n* = 12). C) Representative images of HE staining of LT biopsies from cohort 1 (scale bar = 200 µm). D) Representative images and relative quantification of TUNEL staining in LT biopsies from cohort 1 (scale bar = 200 µm). E) ROS, MDA, GSH, and SOD levels in LT biopsies from cohort 1 (HLA group, n = 18; CTL group, *n* = 12). F) Representative images of Prussian blue staining of LT biopsies from cohort 1 (scale bar = 50 µm). G) Iron content in LT biopsies from cohort 1 (HLA group, *n* = 18; CTL group, *n* = 12). H) Western blot analysis of ferroptosis‐associated protein levels in LT biopsies before and after reperfusion. I) Schematic diagram of the drug treatment experiment utilizing hepatic IRI model mice. J) Serum AST and ALT levels in mice (*n* = 7/group) treated with/without lactate following IRI. K) Representative images of HE‐stained liver tissues from mice treated with/without lactate following IRI (scale bar = 100 µm). L) Western blot analysis of ferroptosis‐associated protein levels in liver tissues from mice treated with/without lactate following IRI. M) Representative TEM images of liver tissues from mice treated with/without lactate following IRI (scale bar = 2 µm). N) Representative images and relative quantification of TUNEL staining in MPH and THLE2 cells treated with/without lactate following H/R (scale bar = 200 µm). O) ROS, MDA, GSH, and SOD levels in MPH cells treated with/without lactate following H/R. For all the above experiments, the data are presented as the means ± SDs. For N‒O, 3 independent experiments (*n* = 3) with similar results were performed in triplicate. In A, D, E, G, J, N, and O, the statistical analyses were performed via two‐tailed unpaired Student's t‐tests. *p* < 0.05 was considered to indicate statistical significance.

To investigate the relationship between hyperlactatemia and hepatic IRI, we first assessed the correlation between serum lactate levels and liver injury in a cohort of adult patients undergoing LT (*n* = 30). The patients were divided into a hyperlactatemia (HLA) group (> = 2.1 mmol L^−1^, *n* = 18) and a control (CTL) group (< 2.1 mmol L^−1^, *n* = 12) according to whether they experienced hyperlactatemia during LT surgery. Compared with the CTL group, the HLA group presented higher serum alanine aminotransferase (ALT) and aspartate aminotransferase (AST) levels at postoperative day (POD)1 (**Figure** **1**A) and slower recovery of serum ALT and AST levels from POD1 to POD7 (Figure 1B). Moreover, liver biopsies from the HLA group presented more obvious alterations in liver histology and increased hepatocellular death, as shown by hematoxylin‒eosin (HE) staining (Figure 1C) and TUNEL staining (Figure 1D).

Next, we investigated whether hyperlactatemia exacerbates ferroptosis during LT surgery. Increased lipid peroxidation is an essential feature of ferroptosis.^[^ ^15^ ^]^ We measured the levels of ROS and malondialdehyde (MDA), indicators of oxidative stress, as well as the contents of glutathione (GSH) and superoxide dismutase (SOD), two crucial antioxidant factors, and found greater lipid peroxidation in the HLA group than in the CTL group (Figure 1E). The accumulation of iron, another important factor that triggers ferroptosis,^[^ ^15^ ^]^ was significantly greater after reperfusion in the HLA group than in the CTL group (Figure 1F,G). In addition, multiple hallmarks associated with ferroptosis were analyzed, and the protein levels of nuclear factor erythroid‐derived 2‐like 2 (NRF2), solute carrier family 7 member 11 (SLC7A11), and glutathione peroxidase 4 (GPX4), which can inhibit ferroptosis, were dramatically lower after reperfusion in the HLA group than in the CTL group; however, the expression levels of the ferroptosis‐promoting proteins cyclooxygenase 2 (COX2), acyl‐CoA synthetase long‐chain family member 4 (ACSL4) and transferrin receptor (TFRC) were greater in the HLA group than in the CTL group (Figure 1H). We thus speculate that hyperlactatemia may aggravate hepatic IRI through ferroptosis.

To test this hypothesis, we established a lactate‐pretreated mouse hepatic IRI model (Figure 1I, top). The in vivo results confirmed that the livers of the mice in the HLA group were more susceptible to IRI than those of mice in the CTL group, as evidenced by higher serum ALT and AST levels (Figure 1J), poorly preserved hepatic architecture (Figure 1K), and increased cell death (Figure S1A, Supporting Information). Consistent with the clinical observations, Prussian blue staining (Figure S1B, Supporting Information), iron content analysis (Figure S1C, Supporting Information), lipid peroxidation analysis (Figure S1D), and Western blotting (Figure 1L) further demonstrated that ferroptosis was more severe in the livers of mice in the HLA group than in those of mice in the CTL group. Transmission electron microscopy (TEM) is frequently used to detect alterations in the morphology of mitochondria associated with ferroptosis.^[^ ^15^ ^]^ Importantly, TEM revealed that, compared with those of mice in the CTL group, the livers of mice in the HLA group exhibited ferroptosis during IRI, with smaller mitochondria and fewer or no mitochondrial cristae (Figure 1M).

We further used mouse primary hepatocytes (MPHs) and THLE2 cells to validate the above results. Figure S1E,F (Supporting Information) illustrates the protocol of MPH extraction as well as the method used to construct a cellular hypoxia‒reoxygenation (H/R) injury model. TUNEL staining (Figure 1N) and calcein‐acetoxymethyl (Calcein‐AM)/propidium iodide (PI) double‐staining (Figure S1G, Supporting Information) confirmed that hepatocytes cultured under high‐lactate conditions exhibited increased cell death during H/R. Lipid peroxidation analysis (Figure 1O; Figure S1H, Supporting Information) and Western blotting (Figure S1I, Supporting Information) further confirmed that hepatocytes cultured under high‐lactate conditions exhibited increased ferroptosis during H/R. Consistent with these findings, TEM revealed an increased mitochondrial membrane density, fragmented mitochondrial outer membranes, and disrupted mitochondrial ridges in hepatocytes cultured with high lactate levels compared with hepatocytes cultured under normal conditions (Figure S1J, Supporting Information).

Furthermore, we investigated the role of ferroptosis in hyperlactatemia‐mediated hepatic IRI. Mice with hyperlactatemia were treated with ferrostatin‐1 (Fer‐1), a selective inhibitor of ferroptosis (Figure 1I, bottom). The results indicated that Fer‐1 markedly attenuated hepatic IRI in mice with hyperlactatemia, as shown by reduced serum transaminase levels (Figure S2A, Supporting Information), relatively well‐preserved hepatic architecture (Figure S2B, Supporting Information), and decreased cell death (Figure S2C, Supporting Information). Moreover, Fer‐1 markedly reduced the accumulation of iron (Figure S2D, Supporting Information) and lipid peroxidation (Figure S2E, Supporting Information) in the livers of mice with hyperlactatemia during IRI. Data from in vitro studies also validated the effect of Fer‐1 in attenuating hyperlactatemia‐mediated hepatic IRI. TUNEL staining (Figure S2F, Supporting Information), calcein‐AM/PI double staining (Figure S2G, Supporting Information), lipid peroxidation analysis (Figure S2H, Supporting Information), and Western blotting (Figure S2I, Supporting Information) revealed that treating hepatocytes with Fer‐1 to inhibit ferroptosis dramatically ameliorated H/R‐induced damage to hepatocytes cultured under high‐lactate conditions. Taken together, these data demonstrate that lactate exacerbates hepatic IRI through ferroptosis.

### 2.2 Lactate Induces the Lactylation of Mitochondrial PCK2 at K100 During Hepatic IRI

**Figure 2:**
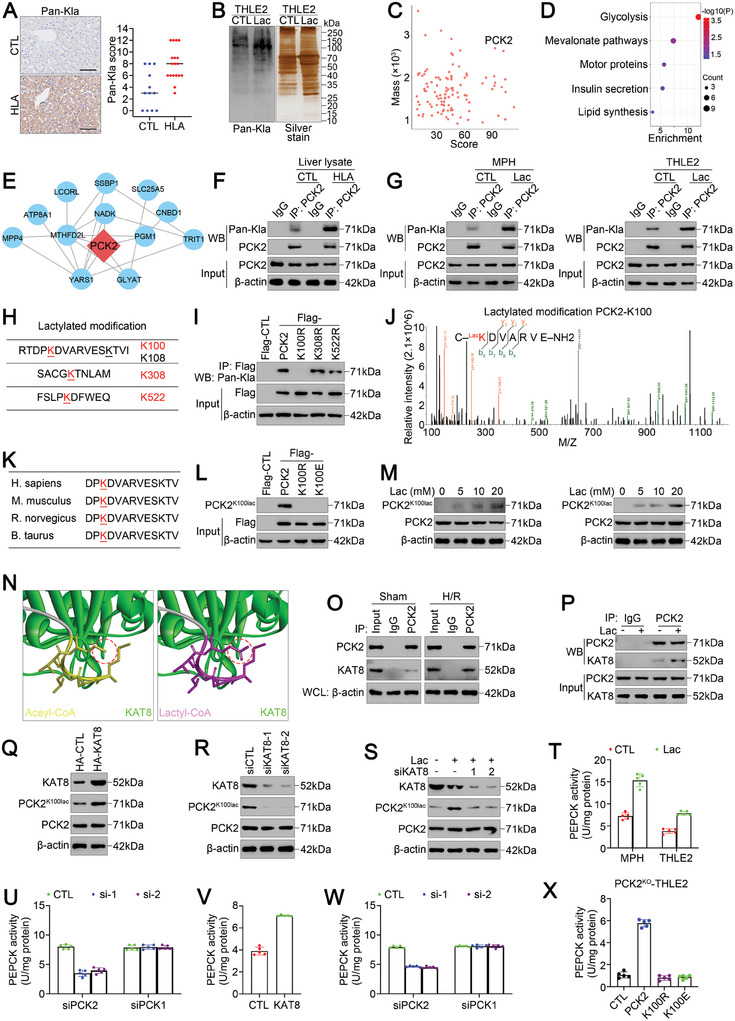
Lactate facilitates the lactylation of PCK2 at K100 via KAT8. A) Representative images of IHC staining with an anti‐pan‐Kla antibody in LT biopsies (scale bar = 100 µm). B) Western blot analysis of pan‐Kla expression and silver staining of lysates from THLE2 cells treated with/without lactate following H/R. C) Scatter plot of global lysine lactylation changes in THLE2 cells treated with/without lactate following H/R. D) KEGG pathway enrichment analysis of lysine‐lactylated proteins in THLE2 cells treated with/without lactate following H/R. E) Protein‒protein interaction network analysis of ferroptosis‐related lysine‐lactylated proteins via the STRING database. F) LT biopsies from patients with/without hyperlactatemia were collected for IP with an anti‐pan‐Kla antibody, followed by Western blotting. G) MPHs and THLE2 cells treated with/without lactate following H/R were collected for IP with an anti‐pan‐Kla antibody, followed by Western blotting. H) Potential lactylation sites of PCK2. I) After H/R exposure, lysates of THLE2 cells expressing Flag‐CTL, Flag‐PCK2, Flag‐PCK2 K100R, Flag‐PCK2 K308R, or Flag‐PCK2 K522R and cultured under high‐lactate conditions were incubated with an anti‐Flag antibody, and interacting proteins were detected with an anti‐pan‐Kla antibody via Western blotting as indicated. J) LC–MS/MS analysis of modified (Kla)DVARVE is shown. K) The Lys100 residue of PCK2 is highly conserved in different species, ranging from *M. musculus* to *H. sapiens*. L) Lysates of HEK293T cells expressing Flag‐CTL, Flag‐PCK2, Flag‐PCK2 K100R, and Flag‐PCK2 K100E were incubated with an anti‐Flag antibody, and interacting proteins were detected with an anti‐PCK2^K100lac^ antibody via Western blotting as indicated. M) After H/R exposure, lysates of MPHs and THLE2 cells cultured with different concentrations of lactate were incubated with an anti‐PCK2^K100lac^ antibody for Western blotting. N) The cofactor pocket of KAT8 is bound to acetyl‐CoA (left) and to lactyl‐CoA (right). The transfer groups in acetyl‐CoA and lactyl‐CoA are indicated with red circles. O) Before and after reperfusion, MPH lysates were incubated with an anti‐PCK2 antibody, and interacting proteins were detected with an anti‐KAT8 antibody via Western blotting as indicated. WCL (Whole Cell Lysate) P) After H/R exposure, lysates of THLE2 cells cultured with/without lactate were incubated with an anti‐PCK2 antibody, and interacting proteins were detected with an anti‐KAT8 antibody via Western blotting as indicated. Q) Western blot analysis of KAT8, PCK2^K100lac^, and PCK2 expression in THLE2 cells expressing HA‐CTL or HA‐KAT8. R) Western blot analysis of KAT8, PCK2^K100lac^, and PCK2 expression in THLE2 cells transfected with siCTL or siKAT8. S) Western blot analysis of KAT8, PCK2^K100lac^, and PCK2 expression in THLE2 cells cultured with/without lactate and transfected with/without siKAT8, as indicated. T) PEPCK activity in THLE2 cells treated with/without lactate following H/R. U) PEPCK activity in THLE2 cells transfected with PCK2 siRNA or PCK1 siRNA and treated with lactate following H/R. V) PEPCK activity in THLE2 cells transfected with HA‐KAT8 following H/R. W) PEPCK activity in THLE2 cells transfected with HA‐KAT8 with or without PCK1/2 siRNA following H/R. X) PEPCK activity in KAT8‐overexpressing PCK2^KD^ THLE2 cells transfected with Flag‐PCK2, Flag‐PCK2 K100R or Flag‐PCK2 K100E following H/R. For all the above experiments, the data are presented as the means ± SDs. Three independent experiments (*n* = 3) with similar results were performed in triplicate. In A and T‐X, the statistical analyses were performed via two‐tailed unpaired Student's t‐tests. *p* < 0.05 was considered to indicate statistical significance.

Lactate can alter protein expression, localization, or function via lysine lactylation (Kla).^[^ ^16^ ^]^ Using an anti‐pan‐Kla antibody, we observed that the global levels of lactylated lysine were substantially greater in the liver tissues subjected to hyperlactatemia during LT surgery (**Figure** **2**A), which was validated in lactate‐pretreated hepatic IRI models in vitro (Figure 2B; Figure S3A, Supporting Information) and in vivo (Figure S3B, Supporting Information). To gain comprehensive insight into hyperlactatemia‐mediated ferroptosis‐related lactylation, we used liquid chromatography‒tandem mass spectrometry (LC‒MS/MS) analysis to investigate lysine‐lactylated substrates in THLE2 cells treated with/without lactate following H/R. Notably, we identified 112 lysine lactylation sites across 75 proteins (Table S1, Supplementary Table), with significantly increased lactylation levels at 107 lysine lactylation sites on 72 proteins (fold change ≥ 2, *p* < 0.05) (Figure 2C). We subsequently performed Kyoto Encyclopedia of Genes and Genomes (KEGG) functional enrichment analysis on the proteins with increased Kla levels. Compared with hepatocytes exposed to H/R alone, the proteins that exhibited increased Kla in hepatocytes cultured under high‐lactate conditions and exposed to H/R were enriched in ferroptosis‐related metabolic pathways, such as glycolysis, the mevalonate pathway and lipid synthesis (Figure 2D). We further analyzed the ferroptosis network associated with the substrates that exhibited increased Kla levels via the Search Tool for the Retrieval of Interacting Genes/Proteins (STRING) database. Among the ferroptosis‐related proteins identified, mitochondrial PCK2 is the rate‐limiting enzyme that catalyzes the production of glucose from lactate (Figure 2E); however, its role in ferroptosis during hepatic IRI remains unknown. Importantly, H/R injury in THLE2 cells cultured under high‐lactate conditions was markedly attenuated when PCK2 was depleted (Figure S2C,D, Supporting Information). Thus, we focused on PCK2 lactylation.

We immunoprecipitated total protein extracts from liver tissues or hepatocytes with an anti‐PCK2 antibody, followed by Western blotting with an anti‐pan‐Kla antibody. Indeed, PCK2 was lactylated in hepatocytes, and PCK2 lactylation was markedly increased in the livers of lactate‐pretreated mice (Figure 2F) and hepatocytes cultured under high‐lactate conditions (Figure 2G). LC–MS/MS analysis revealed four lactylation sites in the PCK2 protein sequence, but there was a significant increase in lactylation at only three lactylation (red) sites after lactate treatment (Figure 2H), implying that the lactylation of these three sites could be regulated in hepatocytes. To identify the critical lactylation site of PCK2 in hyperlactatemia‐mediated ferroptosis during hepatic IRI, lactylation‐defective mutants were generated for these three lactylation sites by substituting K for R. We found that only the PCK2 K100R mutant resulted in reduced lactylation in THLE2 cells cultured under high‐lactate conditions (Figure 2I). The b‐y ion matching diagram for PCK2 K100 is shown in Figure 2J. Notably, the K100 residue of PCK2 is highly conserved in different species, ranging from *Homo sapiens* to *Mus musculus* (Figure 2K). To further confirm that PCK2 is lactylated at K100 during hepatic IRI, an antibody that specifically recognizes PCK2 lactylated at K100 (PCK2^K100lac^) was generated, and its specificity was verified via immunohistochemistry (IHC) assays (Figure S3E, Supporting Information) and in cells expressing PCK2‐WT, ‐K100R, or ‐K100E (Figure 2L). Indeed, lactate treatment strongly induced PCK2 lactylation at K100 (Figure 2M), indicating that the K100 residue of PCK2 is an essential lactylation site in hyperlactatemia‐mediated ferroptosis during hepatic IRI. These results indicate that lactate induces mitochondrial PCK2 lactylation at K100 during hepatic IRI.

### 2.3 KAT8 Lactylates PCK2 at K100 and Promotes PCK2 Activity

Next, we investigated the potential lysine lactyltransferase that lactylates PCK2. Proteins that interact with PCK2 were captured from THLE2 cell lysates via an anti‐PCK2 antibody and analyzed via LC‒MS/MS to generate a PCK2‐binding protein library (Table S2, Supporting Information). In addition to previously reported PCK2‐binding proteins, including heat shock protein family D member 1 (HSPD1)^[^ ^17^ ^]^ and RAF1,^[^ ^18^ ^]^ KAT8 was identified as a novel binding partner of PCK2. KAT8, a member of the MYST protein family of histone acetyltransferases, was recently reported to be responsible for the Kla of protein substrates involved in diverse biological processes.^[^ ^19^ ^]^

To investigate the interaction between PCK2 and KAT8, we docked lactyl coenzyme A (lactyl‐CoA) into the structure of KAT8. Lactyl‐CoA fits well in the cofactor pocket of KAT8, with the structure of the lactyl‐coA–KAT8 complex resembling that of the acetyl coenzyme A (acetyl‐CoA)–KAT8 complex (Figure 2N). Endogenous PCK2‐KAT8 protein binding was validated in hepatocytes in the presence and absence of H/R (Figure 2O; Figure S3F, Supporting Information). Coimmunoprecipitation (CoIP) and Western blot analysis of HEK293T cells revealed that reconstituted Flag‐PCK2 directly interacted with HA‐tagged KAT8 (Figure S3G, Supporting Information). We then investigated whether lactate influences the interaction between KAT8 and PCK2. Strikingly, lactate treatment slightly increased the binding of KAT8 and PCK2 after reoxygenation (Figure 2P). Moreover, PCK2 lactylation at K100 increased when KAT8 was overexpressed (Figure 2Q), whereas it was suppressed by KAT8 knockdown (Figure 2R). Moreover, lactate‐induced lactylation of PCK2 at K100 was inhibited by KAT8 knockdown (Figure 2S). Collectively, these results indicate that KAT8 interacts with and lactylates PCK2 at K100.

We next investigated the effect of KAT8 on the regulation of PCK2 expression and activity. Notably, exogenous overexpression or knockdown of KAT8 did not affect the amount of PCK2 protein expressed (Figure 2Q,R). Lactate also failed to affect PCK2 protein expression (Figure 2S), suggesting that KAT8 expression or lactate exposure does not directly affect PCK2 protein levels in hepatocytes. Interestingly, we found that lactate treatment promoted phosphoenolpyruvate carboxykinase (PEPCK) activity in MPH and THLE2 cells after reoxygenation (Figure 2T). We next established stable PCK2 knockdown (PCK2^KD^) THLE2 cells (Figure S3H, Supporting Information). PCK2 knockdown affected cell activity (Figure S3I, Supporting Information) but did not directly result in cell death (Figure S3J, Supporting Information), which was consistent with previous reports.^[^ ^20^ ^]^ Notably, PCK2 knockdown, but not PCK1 knockdown, markedly suppressed the activation of PEPCK by lactate in THLE2 cells (Figure 2U). Similarly, KAT8 overexpression increased PEPCK activity in THLE2 cells after reoxygenation (Figure 2V), whereas only PCK2 knockdown dramatically inhibited the activation of PEPCK caused by KAT8 overexpression in THLE2 cells (Figure 2W). Furthermore, KAT8 overexpression increased PEPCK activity in PCK2‐overexpressing PCK2^KD^ THLE2 cells but not in PCK2 K100R‐ or PCK K100E‐overexpressing PCK2^KD^ THLE2 cells (Figure 2X). Taken together, these results indicate that KAT8 promotes PCK2 activity through lactylation at K100 during hepatic IRI.

### 2.4 PCK2 Renders the Liver Susceptible to Ferroptosis and IRI

**Figure 3:**
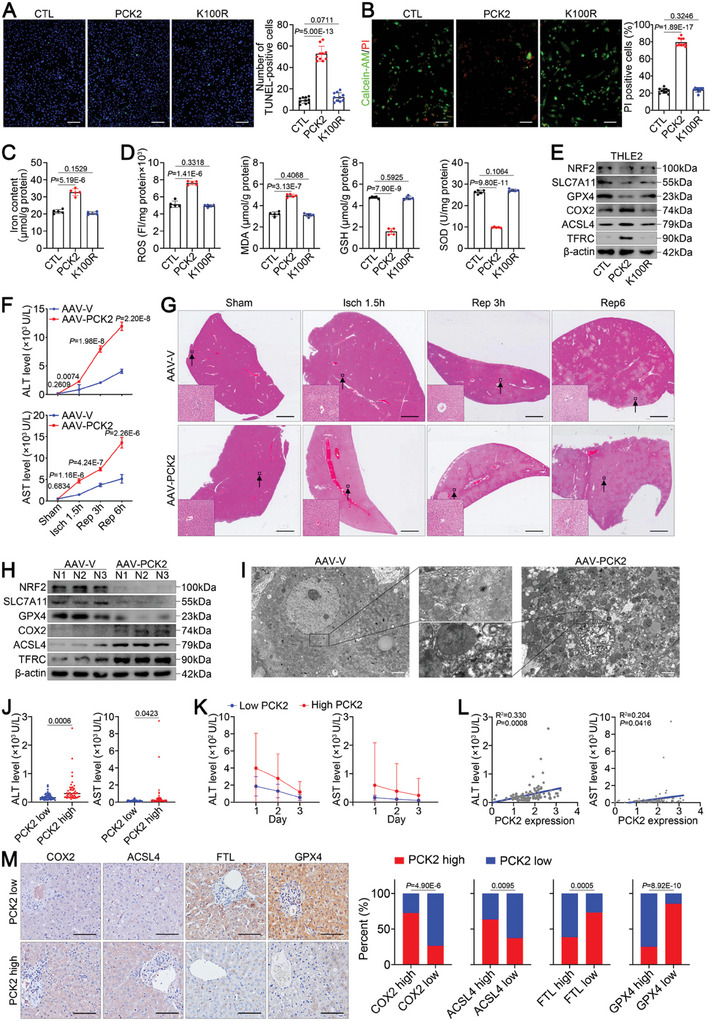
PCK2 renders the liver susceptible to ferroptosis during IRI. A–E) THLE2 cells were transfected with Flag‐CTL, Flag‐PCK2, or Flag‐PCK2 K100R and subjected to H/R. (A) Representative images and relative quantification of TUNEL staining (scale bar = 200 µm). (B) Representative images and relative quantification of calcein‐AM/PI double‐staining (scale bar = 100 µm). (C) Iron content. (D) ROS, MDA, GSH and SOD levels. (E) Western blot analysis of ferroptosis‐associated protein expression. F–I) Hepatic IRI in AAV‐V or AAV‐PCK2‐treated mice (*n* = 7/group). (F) Serum AST and ALT levels at the indicated times. (G) Representative images of HE‐stained livers at the indicated times (scale bar = 1 mm). (H) Western blot analysis of ferroptosis‐associated protein expression. (I) Representative TEM images of liver tissues after reperfusion for 3 h (scale bar = 2 µm). J) Serum AST and ALT levels in LT recipients in cohort 2 on POD1 (low‐PCK2 group, *n* = 52; high‐PCK2 group, *n* = 52). K) The curve of serum ALT and AST levels in LT recipients in cohort 2 from POD1 to POD3 (low‐PCK2 group, *n* = 52; high‐PCK2 group, *n* = 52). L) Pearson correlation analysis between PCK2 expression in donor's livers and the serum ALT or AST levels of recipients in cohort 2 on POD1 (*n* = 100). M) Representative images and correlation analysis of IHC staining of COX2, ACSL4, FTL, and GPX4 in the donor livers of cohort 2 (scale bar = 100 µm). For all the above experiments, the data are presented as the means ± SDs. Three independent experiments (*n* = 3) with similar results were performed in triplicate. The statistical analyses were performed via two‐tailed unpaired Student's t‐tests (A‐D, F, J) and chi‐square tests (M). *p* < 0.05 was considered to indicate statistical significance.

To further investigate whether PCK2 mediates ferroptosis during hepatic IRI, we performed in vitro assays using THLE2 cells. The results demonstrated that overexpression of PCK2, but not PCK2 K100R, dramatically aggravated hepatocyte damage during H/R, as shown by TUNEL staining (**Figure** **3**A) and calcein‐AM/PI staining (Figure 3B). Moreover, quantitative analysis and Western blotting revealed that PCK2 overexpression in hepatocytes markedly promoted the accumulation of intracellular iron (Figure 3C) and lipid peroxidation (Figure 3D), which was accompanied by an increase in the expression of ferroptosis‐inducing proteins and a decrease in the expression of ferroptosis‐inhibiting proteins (Figure 3E) during H/R. The inhibition of KAT8 acetyltransferase reversed these changes, including reducing hepatocyte damage (Figure S4A,B, Supporting Information) and decreasing susceptibility to ferroptosis (Figure S4C–E, Supporting Information) during H/R. These results suggest that KAT8‐mediated lactylation of PCK2 at K100 exacerbates hepatic ferroptosis and IRI.

To further validate the role of ferroptosis in PCK2‐mediated aggravation of hepatic IRI, mice were intravenously injected with a PCK2‐expressing adeno‐associated virus‐8 (AAV8) to generate a mouse strain in which the liver consistently highly expresses PCK2 (AAV‐PCK2). An empty vector (AAV‐V) was used as a negative control. The quantitative real‐time polymerase chain reaction (qRT‒PCR) (Figure S4F, Supporting Information), Western blot (Figure S4G, Supporting Information), and IHC results (Figure S4H, Supporting Information) collectively confirmed the ectopic expression of PCK2 in AAV‐PCK2 mice. A mouse hepatic IRI model was subsequently constructed. Elevated serum transaminase levels (Figure 3F) and increased necrotic areas (Figure 3G) indicated that PCK2 overexpression substantially aggravated hepatic IRI, most notably after 6 h of reperfusion. Moreover, the results of Prussian blue staining (Figure S4I, Supporting Information), iron content analysis (Figure S4J, Supporting Information), lipid peroxidation analysis (Figure S4K, Supporting Information), Western blotting (Figure 3H), and TEM (Figure 3I) confirmed that PCK2 markedly exacerbated hepatic ferroptosis during IRI. Additionally, we administered Fer‐1 to AAV‐PCK2 mice and found that Fer‐1 significantly ameliorated hepatocyte IRI and ferroptosis in AAV‐PCK2 mice, as evidenced by changes in serum transaminase levels (Figure S4L, Supporting Information), liver histology (Figure S4M, Supporting Information), intracellular iron accumulation (Figure S4N‐O, Supporting Information), lipid peroxidation (Figure S4P, Supporting Information) and ferroptosis‐related protein expression (Figure S4Q, Supporting Information), which further indicated that PCK2 aggravates hepatic IRI through ferroptosis.

Next, we analyzed the clinical correlation between PCK2 levels in donor livers and hepatic IRI after LT. We utilized 1:1 propensity score matching (PSM) analysis to exclude cases in which clinical factors affected the recovery of LT recipients and enrolled 100 patients, who were then divided into 2 groups on the basis of the median value of PCK2 expression (TableS3, Supporting Information). Patients who received a donor liver with high PCK2 expression had significantly higher serum ALT and AST levels on POD1 (Figure 3J; Table S4, Supporting Information), as well as slower recovery of AST and ALT levels from POD1‐POD3 (Figure 3K). Further correlation analysis suggested that PCK2 levels in donor's livers were significantly positively correlated with ALT and AST levels in recipients on POD1 (Figure 3L). We further assessed the clinical association between PCK2 expression and ferroptosis susceptibility in donor's livers. Using qRT‒PCR analysis, we detected a significant negative correlation between PCK2 expression and ferritin heavy chain 1 (FTH) and ferritin light chain (FTL) expression (Figure S4R, Supporting Information), indicating that the iron reserve capacity is reduced in livers with high PCK2 expression. Moreover, PCK2 expression tended to be significantly negatively correlated with glutathione peroxidase 4 (GPX4) expression (Figure S4S, Supporting Information), further suggesting that patients with high PCK2 expression have a reduced hepatic antioxidant capacity. Most importantly, the data revealed a significant positive correlation between the expression of PCK2 and that of the ferroptosis marker COX2 (Figure S4T, Supporting Information). Consistent with these results, IHC analysis of donor's livers further confirmed that the PCK2 level was positively correlated with the expression of the ferroptosis drivers COX2 and ACSL4 and strongly inversely correlated with the ferroptosis suppressors GPX4 and FTL (Figure 3M). These data suggest that high PCK2 expression may exacerbate hepatic ferroptosis and IRI.

### 2.5 PCK2 Reprograms OXSM‐Mediated mtFAS

**Figure 4:**
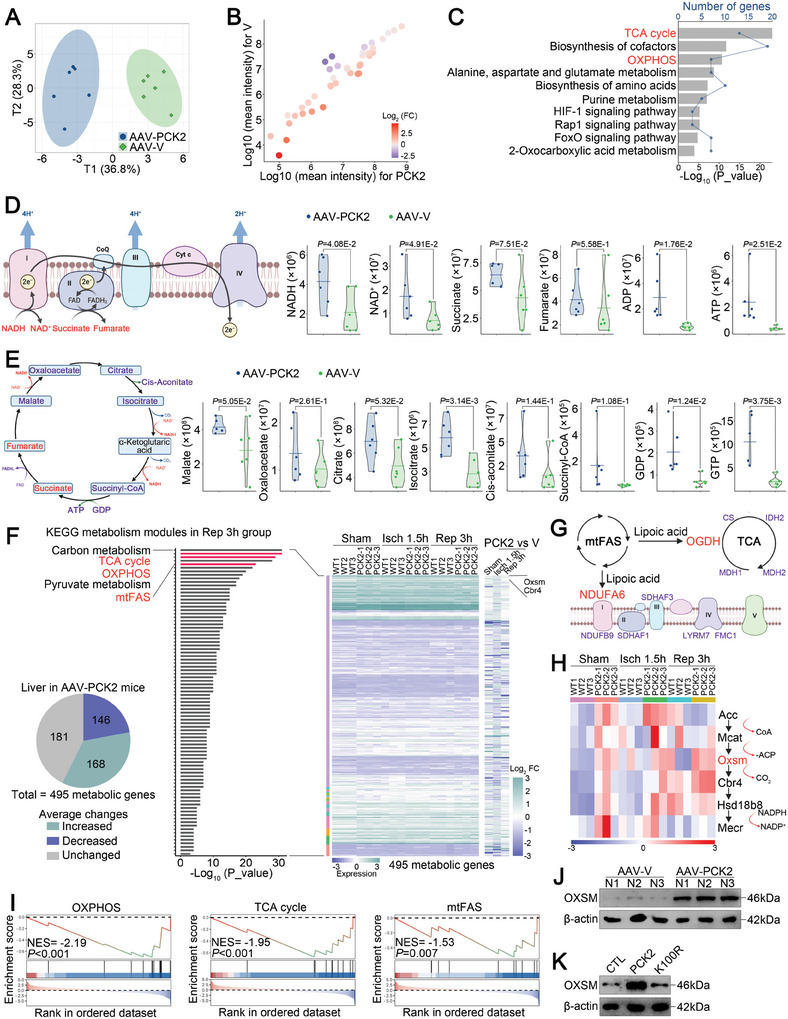
Metabolomic and transcriptomic analyses revealed altered mtFAS and OXSM expression in the livers of AAV‐PCK2‐treated mice. A–G) Metabolite levels in the livers of AAV‐PCK2‐ and AAV‐V‐treated mice were analyzed by LC‐MS (*n* = 6 samples/group). (A) Dimensionality reduction analysis, the ellipse lines indicate 95% CI ranges with means. (B) Analysis of differentially abundant metabolites in the livers of AAV‐PCK2‐ and AAV‐V‐ treated mice. (C) Pathway analysis of the differentially abundant metabolites in the livers of AAV‐PCK2‐ and AAV‐V‐treated mice. (D) Left, schematic diagram of OXPHOS. Right, relative quantification of differentially abundant metabolites associated with OXPHOS in the livers of AAV‐PCK2‐ and AAV‐V‐treated mice. (E) Left, schematic diagram of the TCA cycle. Right, relative quantification of differentially abundant metabolites associated with the TCA cycle in the livers of AAV‐PCK2‐ and AAV‐V‐treated mice. (F‐I) Bulk RNA‐seq analysis of the livers of AAV‐PCK2‐ and AAV‐V‐treated mice. (F) Metabolism‐related genes with a change in expression >50% were subjected to KEGG pathway enrichment analysis, and plots of the metabolism‐related KEGG modules in which these genes were enriched were generated. (G) Schematic diagram of mtFAS to OXPHOS and the TCA cycle. H) Heatmap of the expression of the top upregulated genes involved in mtFAS. I) GSEA of metabolism‐related genes with a change in expression >50% in the livers of AAV‐PCK2‐ and AAV‐V‐treated mice. J) Western blot analysis of OXSM expression in the livers of AAV‐PCK2‐ and AAV‐V‐treated mice. K) Western blot analysis of OXSM expression in THLE2 cells transfected with Flag‐CTL, Flag‐PCK2, or Flag‐PCK2 K100R and subjected to H/R. For D‐E, the data are presented as the means ± SDs, and statistical analyses were performed via two‐tailed unpaired Student's t‐tests. For J‐K, 3 independent experiments (*n* = 3) with similar results were performed in triplicate. *p* < 0.05 was considered to indicate statistical significance.

PCK2 is involved in hepatic gluconeogenesis.^[^ ^21^ ^]^ To further investigate whether PCK2 regulates hepatic IRI and ferroptosis through metabolic remodeling, we performed a mass spectrometry‐based metabolomic analysis of livers from AAV‐PCK2 mice and AAV‐V mice. Principal component analysis revealed that PCK2 overexpression led to a global metabolic alteration (**Figure** **4**A), with the levels of 23 metabolites being markedly increased and the levels of 7 metabolites being obviously decreased (fold change ≥ 1.5, *p *< 0.05) (Figure 4B). Enrichment analysis of the differentially abundant metabolites revealed an increased abundance of metabolites involved in the TCA cycle, OXPHOS, and amino acid and purine metabolism in the livers of AAV‐PCK2 mice compared with those of the AAV‐V mice (Figure 4C). Specifically, the levels of multiple metabolites associated with OXPHOS (Figure 4D) and the TCA cycle (Figure 4E) were dramatically elevated in the livers of AAV‐PCK2 mice.

To further determine the metabolic gene expression signature of the livers of AAV‐PCK2 mice, we performed RNA sequencing (RNA‐seq) and then assessed the involvement of the differentially expressed metabolism‐related genes in 87 metabolism‐related KEGG pathways. Interestingly, transcriptome analysis revealed that the expression of a substantial portion of metabolism‐related genes was altered in the livers of AAV‐PCK2 mice compared with those of AAV‐V mice during hepatic IRI (Figure 4F, left). Indeed, among the 495 differentially expressed metabolism‐related genes detected, genes related to OXPHOS, the TCA cycle, and mtFAS exhibited the greatest changes in expression (Figure 4F, right). However, the differentially expressed genes were not involved in other pathways in which PCK2 was previously been reported to be involved, specifically gluconeogenesis,^[^ ^21^ ^]^ suggesting that PCK2 may act through a nonclassical mechanism in AAV‐PCK2 mice during hepatic IRI. The mtFAS pathway is essential for the production of lipoic acid, a cofactor required for the maturation of mitochondrial dehydrogenases involved in OXPHOS and the TCA cycle (Figure 4G).^[^ ^13^, ^14^ ^]^ Thus, we speculated that PCK2 mediates multiple pathway alterations via mtFAS and exacerbates ferroptosis during hepatic IRI.

Coincidentally, specific analysis of genes related to the mtFAS pathway revealed that OXSM and carbonyl reductase 4 (CBR4) were the 2 most robustly upregulated metabolism‐related genes after reperfusion (Figure 4H). Moreover, OXSM is a potential PCK2‐interacting protein (Table S2, Supporting Information). Considering that OXSM is a rate‐limiting enzyme that catalyzes the committed step in de novo mtFAS,^[^ ^13^, ^14^ ^]^ we focused subsequent analyses on OXSM. Additionally, gene set enrichment analysis (GSEA) further confirmed that OXPHOS, the TCA cycle, and mtFAS were the top mitochondrial metabolic pathways in which the differentially expressed genes were enriched (Figure 4I), with OXSM expression being significantly upregulated in AAV‐PCK2 mice during hepatic IRI (Figure 4J); this finding that was verified by an increase in OXSM expression at the protein level in vitro (Figure 4K). Collectively, the results of the metabolomic and transcriptomic analyses revealed that OXSM‐mediated mtFAS is a major activated metabolic pathway in PCK2‐induced aggravation of hepatic ferroptosis and IRI.

### 2.6 Lactate Induces PCK2‐Dependent Metabolic Reprogramming During IRI

**Figure 5:**
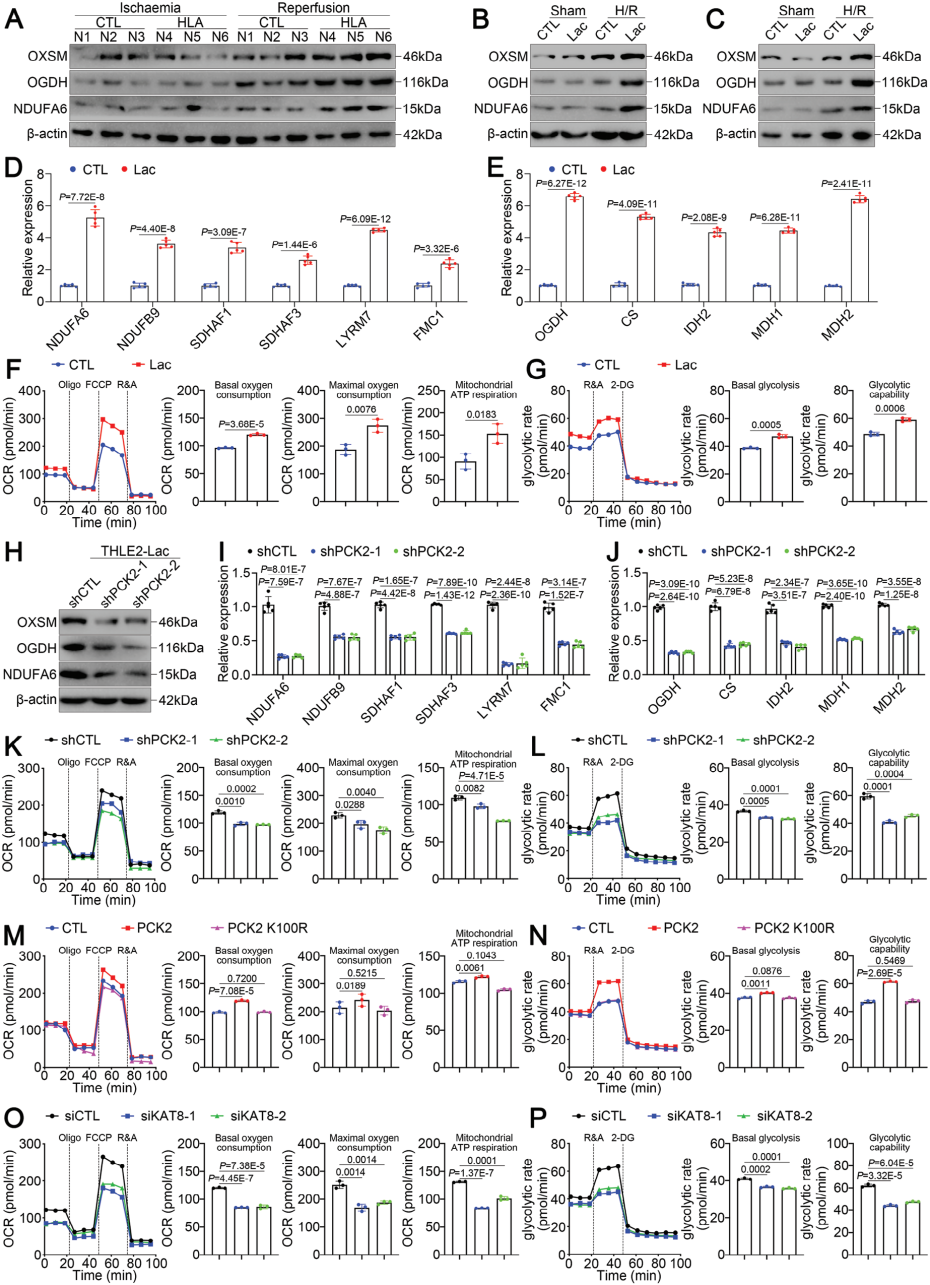
Hyperlactatemia facilitates PCK2‐dependent mtFAS metabolic reprogramming. A) Western blot analysis of OXSM, OGDH, and NDUFA6 expression in LT biopsies before and after reperfusion. B, C) Western blot analysis of OXSM, OGDH, and NDUFA6 expression in (B) MPHs and (C) THLE2 cells treated with/without lactate for 24 h and subjected to H/R. (D‐E) Real‐time qPCR validation of the top significantly downregulated D) OXPHOS‐related genes and E) TCA cycle‐related genes in THLE2 cells treated with/without lactate for 24 h and subjected to H/R. F, G) Real‐time Seahorse OCR and glycolytic rate measurements to evaluate the OXPHOS and TCA cycle rate in THLE2 cells treated with/without lactate for 24 h and subjected to H/R. H) Western blot analysis of OXSM, OGDH, and NDUFA6 expression in THLE2 cells transfected with shCTL or shPCK2, subjected to H/R, and cultured under high‐lactate conditions. I, J) Real‐time qPCR validation of the top significantly downregulated (I) OXPHOS‐related genes and (J) TCA cycle‐related genes in THLE2 cells transfected with shCTL or shPCK2, subjected to H/R, and cultured under high‐lactate conditions. K, L) Real‐time Seahorse OCR and glycolytic rate measurements to evaluate the OXPHOS and TCA cycle rate in THLE2 cells transfected with shCTL or shPCK2, subjected to H/R, and cultured under high‐lactate conditions. M, N) Real‐time Seahorse OCR and glycolytic rate measurements to evaluate the OXPHOS and TCA cycle rate in THLE2 cells transfected with Flag‐CTL, Flag‐PCK2 or Flag‐PCK2 K100R, subjected to H/R, and cultured under high‐lactate conditions. O, P) Real‐time Seahorse OCR and glycolytic rate measurements to evaluate the OXPHOS and TCA cycle rate in THLE2 cells transfected with siCTL or siKAT8, subjected to H/R, and cultured under high‐lactate conditions. For all the above experiments, the data are presented as the means ± SDs. Three independent experiments (*n* = 3) with similar results were performed in triplicate. In D‐G and I‐P, the statistical analyses were performed via two‐tailed unpaired Student's t‐tests. *p* < 0.05 was considered to indicate statistical significance.

Previous work revealed that mtFAS repletes lipoic acid and facilitates OXPHOS and the TCA cycle (Figure 4G),^[^ ^13^, ^14^ ^]^ whereas either OXPHOS or the TCA cycle increases cellular susceptibility to ferroptosis under physiological and pathological conditions.^[^ ^8^ ^]^ We found that lactate failed to dramatically increase the basal levels of OXSM as well as the levels of NDUFA6 and OGDH (**Figure** **5**A), molecular regulators that control OXPHOS and the TCA cycle (Figure 4G), respectively. However, lactate stimulated the expression of OXSM, OGDH, and NDUFA6 (Figure 5A) in the livers of patients after reperfusion. Consistently, lactate pretreatment increased OXSM, OGDH, and NDUFA6 protein levels (Figure S5A, Supporting Information) in the livers of the mice in the IRI group but not in those of mice in the sham group. This finding was also confirmed in hepatocytes, where OXSM, OGDH, and NDUFA6 expression was dramatically increased by lactate stimulation but not under basal conditions after reoxygenation (Figure 5B,C). Consistent with these findings, qRT‒PCR assays further confirmed the significant upregulation of genes (Figure 5D,E) involved in OXPHOS and the TCA cycle (Figure 4G) in lactate‐pretreated hepatocytes. Moreover, real‐time oxygen consumption rate (OCR) and glycolytic rate measurements revealed that, after exposure to H/R, THLE2 cells cultured under high‐lactate conditions presented greater basal oxygen consumption, mitochondrial ATP respiration, and maximal oxygen consumption than did those cultured without lactate (Figure 5F). Moreover, basal glycolysis and glycolytic capability were markedly increased in lactate‐pretreated hepatocytes (Figure 5G), suggesting that lactate markedly potentiated OXPHOS and TCA cycle activity during hepatic IRI.

Next, we investigated whether PCK2 controls mtFAS‐mediated metabolic reprogramming induced by lactate during hepatic IRI. In contrast to those in lactate‐pretreated THLE2 cells expressing shCTL, the protein levels of OXSM, OGDH, and NDUFA6 in PCK2^KD^ THLE2 cells were dramatically lower (Figure 5H). At the mRNA level, the expression of several factors pivotal for OXPHOS and the TCA cycle was consistently downregulated in PCK2^KD^ THLE2 cells (Figure 5I,J). Real‐time OCR and glycolytic rate measurements revealed that even in the presence of lactate, basal oxygen consumption, mitochondrial ATP respiration, maximal oxygen consumption (Figure 5K), basal glycolysis and glycolytic capability (Figure 5L) were decreased in PCK2^KD^ THLE2 cells following H/R, supporting our hypothesis that lactate promotes metabolic reprogramming in a PCK2‐dependent manner.

We further investigated the effect of PCK2 lactylation at K100 on PCK2‐mediated mtFAS metabolic reprogramming. We constructed PCK2‐ or PCK2 K100R‐overexpressing PCK2^KD^ THLE2 cells. As anticipated, after H/R, PCK2‐overexpressing, but not PCK2 K100R‐overexpressing PCK2^KD^ THLE2 cells, presented increased OXSM, OGDH, and NDUFA6 protein levels (Figure S5B, Supporting Information), increased mRNA levels of OXPHOS‐ and TCA cycle‐related genes (Figure S5C,D, Supporting Information), and increased OCRs and glycolytic rates (Figure 5M,N, Supporting Information). Moreover, in THLE2 cells transfected with KAT8 siRNAs (Figure S5E, Supporting Information), downregulated of OXPHOS‐ and TCA cycle‐related genes were also detected (Figure 5O,P; Figure S5E–G, Supporting Information). Collectively, these results reveal an underexplored mechanism by which PCK2 mediates hyperlactatemia‐induced mtFAS metabolic reprogramming.

### 2.7 PCK2 Restricts the Parkin‐Mediated Polyubiquitination of OXSM

**Figure 6:**
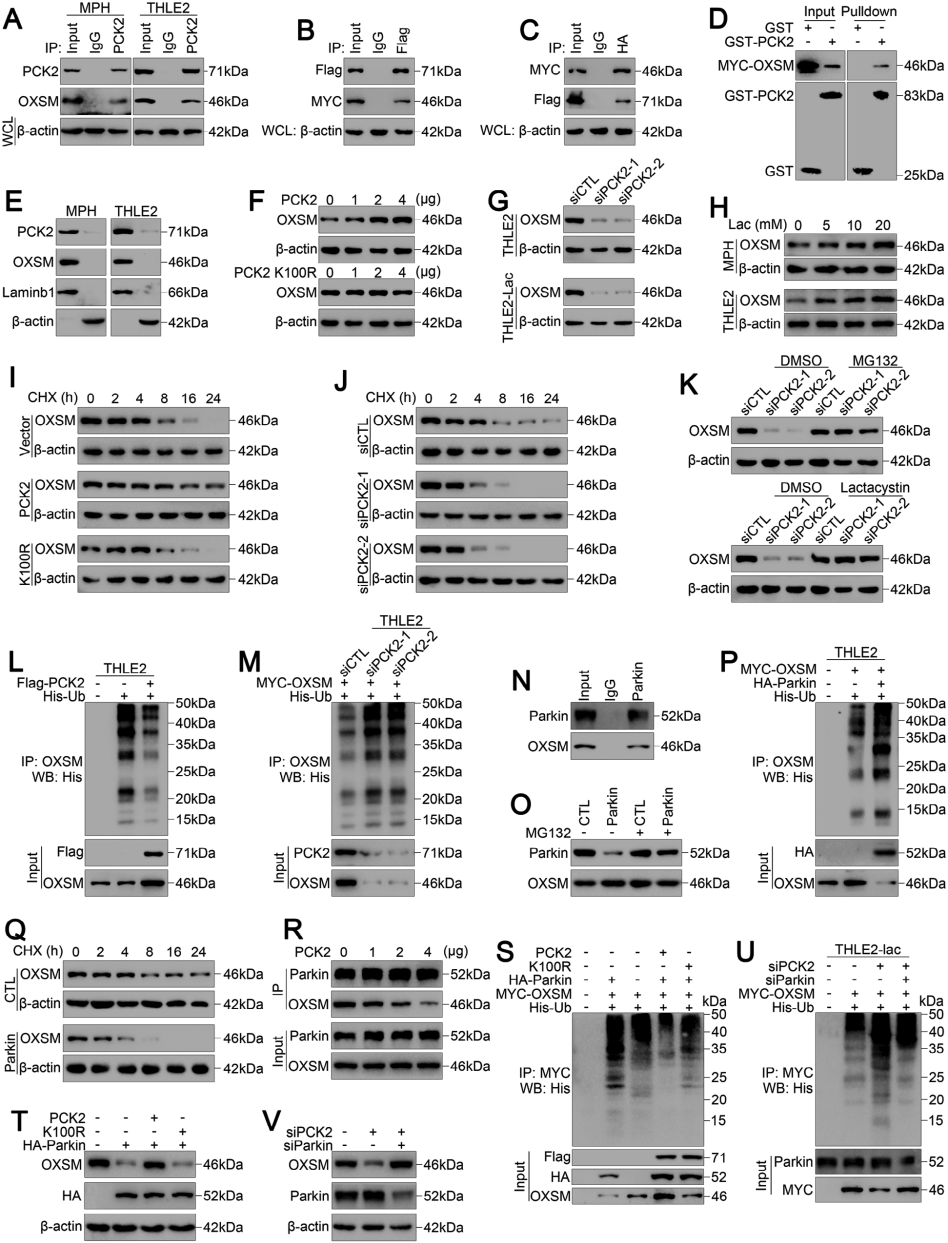
PCK2 abrogates the Parkin‐induced degradation of OXSM. A) MPH and THLE2 cell lysates were incubated with an anti‐PCK2 antibody, and interacting proteins were detected with an anti‐OXSM antibody via Western blotting as indicated. B, C) Lysates of HEK293T cells expressing Flag‐PCK2 and MYC‐OXSM were incubated with an anti‐Flag or anti‐MYC antibody, and interacting proteins were detected with an anti‐MYC or anti‐Flag antibody via Western blotting as indicated. D) GST‐PCK2 or GST was incubated with total lysates of THLE2 cells expressing MYC‐OXSM for 2 hours, and then Western blotting with anti‐MYC or anti‐GST antibodies was performed as indicated. E) The localization of PCK2 and OXSM in MPHs and THLE2 cells was analyzed by immunoblotting. F) Western blot analysis of OXSM expression in THLE2 cells transfected with Flag‐PCK2 or Flag‐PCK2 K100R. G) Western blot analysis of OXSM expression in THLE2 cells transfected with siCTL or siPCK2 and cultured under normal or high‐lactate conditions. H) Western blot analysis of OXSM expression in MPHs and THLE2 cells treated with the indicated concentration of lactate (Lac). I) Western blot analysis of OXSM expression in THLE2 cells transfected with Flag‐CTL, Flag‐PCK2, or Flag‐PCK2 K100R and treated with CHX for the indicated times. J) Western blot analysis of OXSM expression in THLE2 cells transfected with siCTL or siPCK2 and treated with CHX for the indicated times. K) Western blot analysis of OXSM protein expression in the indicated Flag‐PCK2‐overexpressing THLE2 cells transfected with siCTL or siPCK2 and treated with/without MG132 or lactacystin as indicated. L, M) THLE2 cells were transfected as indicated, and the cell lysates were then immunoprecipitated with an anti‐OXSM antibody, and detection was performed with an anti‐His antibody. N) THLE2 cell lysates were incubated with an anti‐Parkin antibody, and interacting proteins were detected with an anti‐OXSM antibody via Western blotting as indicated. O) Western blot analysis of OXSM protein expression in THLE2 cells transfected with HA‐CTL or HA‐Parkin and treated with/without MG132. P) THLE2 cells were transfected as indicated, the cell lysates were then immunoprecipitated with an anti‐OXSM antibody, and detection was performed with an anti‐His antibody. Q) Western blot analysis of OXSM expression in THLE2 cells transfected with HA‐CTL or HA‐Parkin and treated with CHX for the indicated times. R) The interaction between Parkin and OXSM in THLE2 cells transfected with different concentrations of Flag‐PCK2 was evaluated via CoIP assays followed by Western blot analysis. S and U) THLE2 cells were transfected as indicated, and the cell lysates were then immunoprecipitated with an anti‐MYC antibody, and detection was performed with an anti‐His antibody. T and V) Western blot analysis of OXSM protein expression in THLE2 cells transfected with the indicated vectors. For all the above experiments, three independent experiments (*n* = 3) with similar results were performed in triplicate.

Next, we investigated the underlying mechanisms by which PCK2 regulates ferroptosis during hepatic IRI. By treating cells with phosphoenolpyruvate, a direct metabolite of PCK2, we excluded the possibility that PCK2 regulates OXSM, OGDH, and NDUFA6 expression in a metabolism‐dependent manner (Figure S6A, Supporting Information). As previously described, OXSM is a potential PCK2‐binding protein (Table S2, Supporting Information); thus, we analyzed the interaction between PCK2 and OXSM. Indeed, CoIP analysis indicated that PCK2 interacted with endogenous OXSM in hepatocytes (**Figure** **6**A). We also confirmed the interaction between PCK2 and OXSM by coexpressing Flag‐PCK2 and MYC‐OXSM in HEK293T cells (Figure 6B,C). The results of the glutathione‐S‐transferase (GST) pull‐down assay further confirmed the direct interaction between PCK2 and OXSM (Figure 6D). Additionally, cell fractionation analysis revealed that PCK2 and OXSM colocalized in mitochondria (Figure 6E). Taken together, these data indicate that PCK2 physically interacts with OXSM.

Since PCK2 can affect OXSM expression (Figure 5H; Figure S5B, Supporting Information), we investigated how PCK2 modulates OXSM protein levels. Strikingly, PCK2 overexpression barely affected OXSM mRNA levels in vitro and in vivo (Figure S6B,C, Supporting Information). However, overexpression of PCK2 but not PCK2 K100R increased OXSM protein levels in a dose‐dependent manner (Figure 6F). In contrast, the silencing of PCK2 markedly decreased the protein levels (Figure 6G, top) but not the mRNA levels of OXSM (Figure S6D, Supporting Information). Additionally, the protein level (Figure 6H), but not the mRNA level (Figure S6E, Supporting Information), of OXSM was markedly increased in lactate‐pretreated hepatocytes, and silencing of PCK2 reversed the lactate‐induced increase in OXSM protein levels (Figure 6G, bottom). Moreover, overexpression of PCK2 but not PCK2 K100R increased the half‐life of the OXSM protein (Figure 6I), whereas silencing of PCK2 significantly facilitated the degradation of the OXSM protein (Figure 6J) and reversed the impact of lactate on OXSM stability (Figure S6F, Supporting Information).

We then investigated the potential mechanisms by which PCK2 inhibits the degradation of the OXSM protein. Interestingly, the addition of the proteasome inhibitor MG132 effectively abrogated the effects of silencing PCK2 on OXSM protein degradation (Figure 6K, top), but the addition of the lysosome inhibitor chloroquine did not prevent the degradation of the OXSM protein (Figure S6G, Supporting Information). Consistently, the addition of the proteasome inhibitor lactacystin also blocked OXSM protein degradation (Figure 6K, bottom), indicating that PCK2 silencing promoted OXSM degradation via the proteasome pathway.

We next sought to determine whether PCK2 inhibits OXSM degradation by regulating the ubiquitination of OXSM. As anticipated, ectopic expression of PCK2 (Figure 6L), not PCK2 K100R (Figure S6H, Supporting Information), substantially decreased the level of polyubiquitylated OXSM. Conversely, PCK2 knockdown markedly increased OXSM polyubiquitination (Figure 6M). Furthermore, the polyubiquitination of the OXSM protein was markedly inhibited in lactate‐pretreated hepatocytes (Figure S6I, Supporting Information), and silencing of PCK2 robustly reversed the inhibitory effect of lactate on OXSM polyubiquitination in hepatocytes (Figure S6J, Supporting Information). These data illustrate that PCK2 regulates OXSM protein abundance through the ubiquitin‐proteasome pathway.

We then aimed to identify the E3 ligase that mediates the ubiquitination‐mediated degradation of the OXSM protein. We studied the effects of several potential E3 ligases that were previously reported to affect mitochondrial metabolism^[^ ^22^ ^]^ and found that Parkin induced drastic OXSM degradation (Figure S6K, Supporting Information). We then investigated whether Parkin induces the degradation of OXSM and whether PCK2 affects this process. As expected, we found that Parkin interacted with OXSM (Figure 6N) to decrease OXSM protein levels through proteasome‐mediated degradation (Figure 6O), which caused OXSM polyubiquitination (Figure 6P) and thus shorten the half‐life of OXSM (Figure 6Q) in THLE2 cells. These findings imply that Parkin is a critical E3 ligase of OXSM. Importantly, PCK2 overexpression decreased the binding of Parkin to OXSM in a dose‐dependent manner (Figure 6R). Moreover, PCK2 overexpression largely abolished the Parkin‐induced polyubiquitination of the OXSM protein (Figure 6S) and thereby increased the OXSM protein level (Figure 6T). Furthermore, when Parkin expression was presilenced in lactate‐cultured THLE2 cells, silencing of PCK2 failed to promote OXSM polyubiquitination (Figure 6U) and reduce OXSM levels (Figure 6V). These data suggest that PCK2 competitively binds to OXSM and thereby abolishes the interaction of OXSM with the E3 ligase Parkin, which leads to the disruption of the Parkin‐dependent degradation of the OXSM protein.

### 2.8 PCK2‐Mediated Ferroptosis is Dependent on OXSM During Hepatic IRI

**Figure 7:**
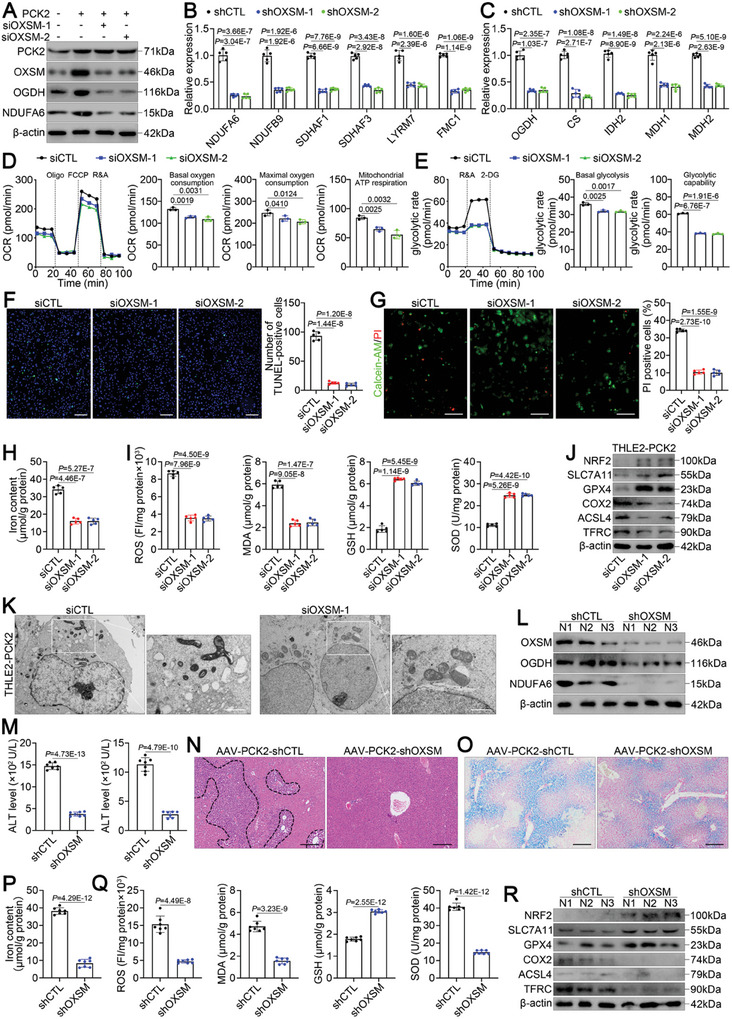
PCK2‐mediated OXSM‐dependent ferroptosis during hepatic IRI. A) Western blot analysis of PCK2, OXSM, OGDH, and NDUFA6 expression in THLE2 cells cotransfected with Flag‐CTL or Flag‐PCK2 and siCTL or siOXSM as indicated. (B‐K) PCK2‐overexpressing THLE2 cells cultured under high‐lactate conditions were transfected with siCTL or siOXSM and subjected to H/R. B, C) Real‐time qPCR analysis of the top significantly downregulated (B) OXPHOS‐related genes and (C) TCA cycle‐related genes. D, E) Real‐time Seahorse OCR and glycolytic rate measurements to evaluate the OXPHOS and TCA cycle rate. F) Representative images and relative quantification of TUNEL staining (scale bar = 200 µm). G) Representative images and relative quantification of calcein‐AM/PI double‐staining (scale bar = 100 µm). H) Iron content. I) ROS, MDA, GSH and SOD levels. J) Western blot analysis of ferroptosis‐associated protein expression. K) Representative TEM images (scale bar = 1 µm). (L‐R) AAV‐PCK2‐treated mice (*n* = 7/group) infected with AAV‐shCTL or AAV‐shOXSM were treated with lactate and subjected to hepatic IRI. L) Western blot analysis of OXSM, OGDH, and NDUFA6 expression in the liver. M) Serum AST and ALT levels. N) Representative images of HE‐stained livers (scale bar = 200 µm). O) Representative images of Prussian blue‐stained livers (scale bar = 200 µm). P) Iron content in the liver. Q) ROS, MDA, GSH, and SOD levels in the liver. R) Western blot analysis of ferroptosis‐associated protein expression in the liver. For all the above experiments, the data are presented as the means ± SDs. Three independent experiments (*n* = 3) with similar results were performed in triplicate. In B‐I, M, P, and Q, the statistical analyses were performed via two‐tailed unpaired Student's t‐tests. *p* < 0.05 was considered to indicate statistical significance.

To determine whether the ferroptosis‐promoting effects of PCK2 are dependent on OXSM during IRI, we inhibited OXSM in PCK2‐overexpressing THLE2 cells and analyzed whether lactate could induce mtFAS. A 90% reduction in OXSM expression induced a significant decrease in OGDH and NDUFA6 expression (**Figure** **7**A) as well as the expression of critical genes involved in OXPHOS (Figure 7B) and the TCA cycle (Figure 7C) in PCK2‐overexpressing THLE2 cells. Moreover, real‐time OCR and glycolytic rate measurements indicated that silencing OXSM in PCK2‐overexpressing THLE2 cells repressed the lactate‐induced increase in basal oxygen consumption, mitochondrial ATP respiration, and maximal oxygen consumption (Figure 7D) as well as basal glycolysis and glycolytic capability (Figure 7E), confirming OXSM dependence. Moreover, TUNEL staining (Figure 7F), calcein‐AM/PI staining (Figure 7G), iron content analysis (Figure 7H), lipid peroxidation analysis (Figure 7I), Western blotting (Figure 7J), and TEM (Figure 7K) further revealed that silencing OXSM markedly inhibited hyperlactatemia‐mediated hepatic IRI and ferroptosis in PCK2‐overexpressing THLE2 cells.

To investigate the role of OXSM in PCK2‐mediated ferroptosis in vivo, we silenced OXSM in the livers of AAV‐PCK2 mice via injection of an AAV9 vector harboring short hairpin RNAs (shRNA) sequences targeting the mouse OXSM gene (hereafter, AAV‐PCK2‐shOXSM mice) and observed an 80% decrease in OXSM protein levels after 4 weeks (Figure 7L). Scramble shRNA was used as a negative control (AAV‐PCK2‐shCTL mice). As expected, despite lactate treatment, OXSM silencing led to reductions in the protein levels of OGDH and NDUFA6 (Figure 7L) as well as the expression of critical genes involved in OXPHOS (Figure S7A, Supporting Information) and the TCA cycle (Figure S7B, Supporting Information). Moreover, OXSM silencing ameliorated hepatic IRI (Figure 7M,N), and inhibited hepatic ferroptosis (Figure 7O,R) in AAV‐PCK2‐shOXSM mice. Overall, these data indicate that the ferroptosis‐promoting effects of PCK2 occur through OXSM during hepatic IRI.

### 2.9 Targeting PCK2 is a Promising Therapeutic Strategy

**Figure 8:**
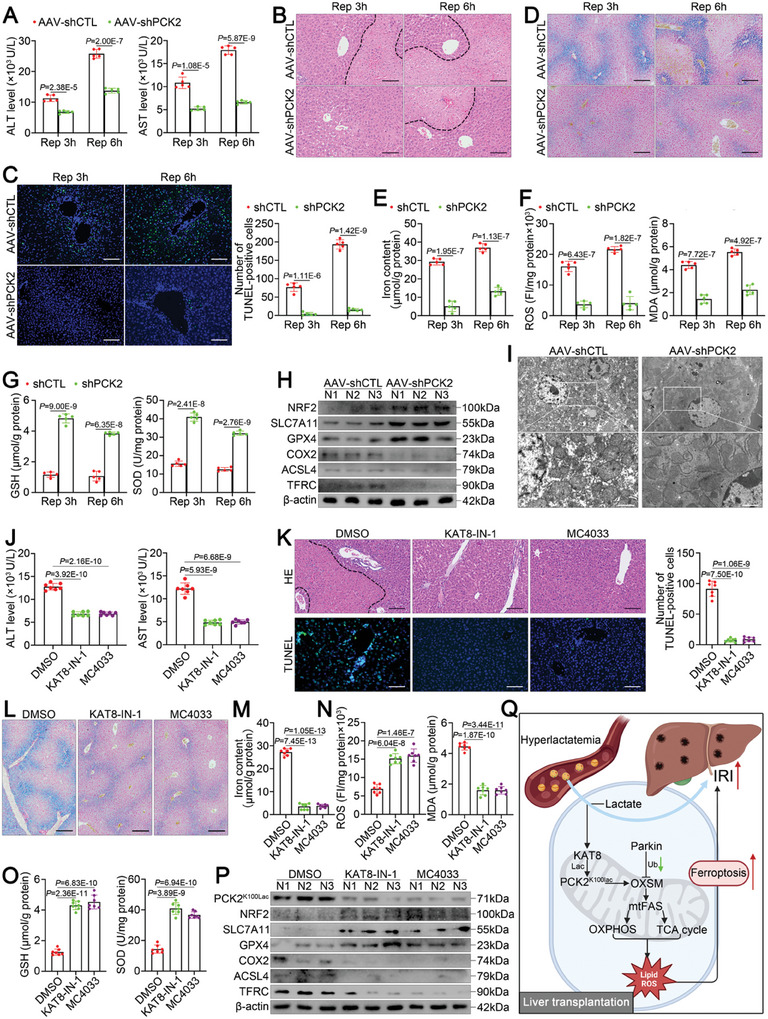
PCK2 is a promising therapeutic target for attenuating hyperlactatemia‐mediated hepatic ferroptosis. A–H) Mice infected with AAV‐shCTL or AAV‐shPCK2 (*n* = 5/group) were treated with lactate and subjected to hepatic IRI. (A) Serum AST and ALT levels at the indicated times. (B) Representative images of HE‐stained livers at the indicated times (scale bar = 100 µm). (C) Representative images of TUNEL‐stained livers at the indicated times and relative quantification of the data (scale bar = 100 µm). (D) Representative images of Prussian blue‐stained livers (scale bar = 200 µm). (E) Iron content in the liver. (F‐G) ROS, MDA, GSH and SOD levels in the liver. (H) Western blot analysis of ferroptosis‐associated protein expression in the liver. I) Representative TEM images of livers (scale bar = 2 µm). J–P) Mice (*n* = 7/group) were treated with the indicated KAT8 inhibitors and lactate and subjected to hepatic IRI. (J) Serum AST and ALT levels. (K) Top, representative images of HE‐stained livers. Bottom, representative images of TUNEL‐stained livers and relative quantification of the data (scale bar = 100 µm). (L) Representative images of Prussian blue‐stained livers (scale bar = 200 µm). (M) Iron content in the liver. (N‐O) ROS, MDA, GSH and SOD levels in the liver. (P) Western blot analysis of ferroptosis‐associated protein expression in the liver. Q) Schematic showing the regulation of hepatic ferroptosis during IRI by lactate, which enhances KAT8‐mediated PCK2 lactylation at K100. This effect diminishes the Parkin‐dependent degradation of OXSM, leading to mtFAS metabolic reprogramming and the potentiation of OXPHOS and TCA cycle activity, thereby exacerbating ferroptosis and IRI in the liver. For all the above experiments, the data are presented as the means ± SDs. In A, C, E‐G, J‐K, and M‐O, the statistical analyses were performed via two‐tailed unpaired Student's t‐tests. *p* < 0.05 was considered to indicate statistical significance.

Currently, numerous strategies have been proposed to attenuate hepatic ferroptosis and IRI,^[^ ^23^ ^]^ but no ferroptosis inhibitors are currently available for clinical application. Given our findings, we investigated whether PCK2 could serve as a potential therapeutic target for the hyperlactatemia‐induced exacerbation of hepatic ferroptosis and IRI. To this end, we performed gene editing or pharmacological interventions in vivo. We first injected an AAV9 vector expressing an shRNA against PCK2 into mice (hereafter, AAV‐shPCK2 mice) and successfully knocked down PCK2 in the liver (Figure S7A–C, Supporting Information), then, we treated the AAV‐shPCK2 mice with lactate prior to hepatic IRI. Indeed, PCK2 knockdown substantially ameliorated hyperlactatemia‐mediated hepatic IRI, as shown by variations in serum transaminase levels (**Figure** **8**A), HE staining (Figure 8B), and TUNEL staining (Figure 8C). Moreover, Prussian blue staining (Figure 8D), iron content analysis (Figure 8E), lipid peroxidation analysis (Figure 8F,G), Western blotting (Figure 8H) and TEM (Figure 8I) further demonstrated that silencing PCK2 markedly inhibited hyperlactatemia‐mediated hepatic ferroptosis during IRI.

In addition to this genetic approach, we used two commercially available, selective KAT8 inhibitors, KAT8‐IN‐1 and MC4033, to pharmacologically and systemically target KAT8. Consistent with the gene knockdown results, measurement of serum transaminase levels (Figure 8J), HE staining (Figure 8K, top), TUNEL staining (Figure 8K, bottom), Prussian blue staining (Figure 8L), iron content analysis (Figure 8M), lipid peroxidation analysis (Figure 8N,O) and Western blotting (Figure 8P) confirmed the ability of KAT8 inhibition to suppress hyperlactatemia‐mediated hepatic IRI and ferroptosis. Importantly, Western blotting revealed that KAT8‐IN‐1 and MC4033 significantly decreased the lactylation of PCK2 at K100 in the liver during IRI (Figure 8P). In conclusion, these results serve as a basis for the clinical translation of therapeutics targeting the KAT8‒PCK2 axis to alleviate IRI in patients with hyperlactatemia undergoing LT.

## 3 Discussion

Owing to disrupted hepatic metabolism, hyperlactatemia is a prominent feature of the perioperative period in LT recipients. Here, we explored the direct links among hyperlactatemia, ferroptosis, and hepatic IRI. As shown in Figure 8Q, hyperlactatemia exacerbates hepatic ferroptosis during IRI by promoting PCK2 activity. Mechanistically, in the context of hyperlactatemia, KAT8 functions as a lysine lactylation transferase and mediates PCK2 lactylation at K100, thereby activating the kinase activity of PCK2 in mitochondrial metabolism. PCK2 subsequently acts as a critical inducer of ferroptosis during IRI by competitively inhibiting the Parkin‐mediated ubiquitination of OXSM, potentiating the production of mtFAS products, and maintaining OXPHOS and TCA cycle activity. Importantly, IRI is a pivotal risk factor for primary graft dysfunction or nonfunction as well as early mortality in patients undergoing LT.^[^ ^3^ ^]^ Our data demonstrate that targeting PCK2 can effectively reduce the expression of OXSM, ultimately inhibiting ferroptosis, thus representing an avenue for effectively alleviating hyperlactatemia‐mediated hepatic IRI in the clinic.

Although various forms of cell death, including apoptosis, necrosis, and pyroptosis, are involved in the development of hepatic IRI, and while numerous targets for these forms of cell death have been proposed, the clinical efficacy of available treatments is currently limited, indicating that new mechanisms of cellular injury remain to be investigated. Ferroptosis is rapidly gaining attention in the field of liver diseases, as excessive iron accumulation is a primary characteristic of most liver diseases, and generally, the liver is vulnerable to oxidative damage. Recent studies have revealed that hyperlactatemia leads to impaired hepatic iron metabolism and systemic iron homeostasis imbalance through increased hepatic hepcidin expression.^[^ ^24^ ^]^ Huang reported that lactate‐mediated upregulation of NADPH‐dependent NOX4 expression contributes to ROS generation and tissue damage.^[^ ^25^ ^]^ However, the association between hyperlactatemia and hepatic ferroptosis has never been investigated. Considering the physiological features of the liver and the factors that induce ferroptosis, we hypothesized that the liver is more susceptible to ferroptosis during IRI when stimulated with lactate. In our study, we comprehensively evaluated the relationships between lactate levels and hepatic IRI, iron metabolism, and oxidative damage. Moreover, treatment with Fer‐1 specifically inhibited ferroptosis and effectively reduced hepatic IRI, demonstrating that the liver is indeed more prone to IRI in a high‐lactate microenvironment. Thus, it is imperative to thoroughly explore the potential mechanisms that regulate ferroptosis to develop effective therapies for combating hyperlactatemia‐mediated exacerbation of IRI.

The current understanding of the function of lactate extends from its origins as a byproduct of glycolysis to its essential role in cellular metabolism as a factor that coordinates signaling among different cells, organs, and tissues.^[^ ^26^ ^]^ Lysine lactylation is a newly discovered posttranslational modification that links gene regulation to cellular metabolism through metabolic dysregulation and epigenetic modifications, affecting the microenvironment and disease development.^[^ ^27^ ^]^ Although the role of lactate in hepatic IRI has not been reported, the involvement of lactylation in tissue injury has been extensively reported. Astrocytic LRP1 enables mitochondrial transfer to neurons and mitigates ischemic stroke by suppressing ARF1 lactylation in the brain.^[^ ^28^ ^]^ Heat shock protein A12A (HSPA12A) maintains aerobic glycolytic homeostasis and histone 3 lactylation in cardiomyocytes to attenuate myocardial IRI.^[^ ^29^ ^]^ Similarly, Du et al. recently revealed that HSPA12A protects the liver from IRI by suppressing glycolysis‐mediated high mobility group box 1 (HMGB1) lactylation and secretion from hepatocytes to inhibit macrophage chemotaxis and inflammatory activation.^[^ ^30^ ^]^ Herein, considering that lactate accumulates in the liver, we first explored the role of lactylation in the regulation of ferroptosis and hepatic IRI. Through proteomics followed by functional enrichment analysis and protein‒protein interaction network analysis, we screened the mitochondrial protein PCK2. Moreover, our data demonstrated that lactate promoted KAT8‐mediated lactylation of PCK2 and increased PCK2 kinase activity, which exacerbated hepatic ferroptosis and IRI. It is believed that PCK2 participates in the pyruvate cycle in glucose‐starved cells and provides carbon for gluconeogenesis and ultimately several downstream anabolic pathways.^[^ ^21^ ^]^ However, through metabolomics and transcriptomics analyses, we observed that, in the livers of AAV‐PCK2 mice subjected to IRI, the pathways altered most substantially were OXPHOS and the TCA cycle, suggesting that PCK2 might regulate hepatic IRI injury through a noncanonical pathway.

The mtFAS pathway, an evolutionarily conserved mitochondrial biosynthetic pathway, generates lipoic acid, which is a required cofactor for several mitochondrial metabolic enzymes involved in OXPHOS and the TCA cycle.^[^ ^13^, ^14^ ^]^ On the basis of the transcriptomic analysis results, we speculated that PCK2 acts on the mtFAS pathway to alter OXPHOS and the TCA cycle, thereby exacerbating ferroptosis during hepatic IRI. Using in vivo and in vitro IRI models, we confirmed that PCK2 alters the expression of OGDH and NDUFA6, which are pivotal metabolic proteins, the OCRs, and the glycolytic rate. Moreover, we confirmed that PCK2 plays a core role in the hyperlactatemia‐induced increase in mtFAS pathway activity and the subsequent increases in OXPHOS and TCA cycle activity, thereby exacerbating hepatic ferroptosis during IRI. Unlike cytosolic fatty acid synthase (FASN), mtFAS is a type II FAS pathway that takes place in the mitochondrial matrix. mtFAS and FASN generate de novo fatty acids via the same chemical process, with the major difference being that in cytosolic FAS, each enzyme is a domain of a single FASN polypeptide rather than a discrete enzyme.^[^ ^13^, ^14^ ^]^ The mitochondrial ketoacyl synthase OXSM is responsible for catalyzing the condensation reaction in the first step of mtFAS and generating ketoacyl intermediates for a series of reduction and dehydration reactions by subsequent enzymes in the mtFAS cycle.^[^ ^13^ ^]^ Our data suggest that PCK2 restricts Parkin‐linked ubiquitination‐mediated degradation of OXSM and that OXSM silencing markedly impairs the activation of PCK2 during mtFAS and downstream activation of the TCA cycle and OXPHOS. Furthermore, our data strongly indicate that mtFAS has the potential as a therapeutic target for hepatic IRI, but no inhibitors are available for clinical application. Here, we attempted to target PCK2 and found that, after inhibiting PCK2 expression or activity, OXSM expression was effectively suppressed, resulting in the inhibition of ferroptosis and markedly alleviating hyperlactatemia‐mediated hepatic IRI.

There are several limitations to this study that should be addressed in future research. PCK2 is a mitochondrial gene involved in glycogen synthesis and is specifically expressed at high levels in hepatocytes,^[^ ^31^ ^]^ we thus focused on the role of PCK2 mainly in hepatocytes. It has been extensively reported that multiple factors in the immune microenvironment, such as macrophages, induce ferroptosis,^[^ ^32^ ^]^ but whether lactate exacerbates hepatic ferroptosis and IRI via immune cells requires further investigation. In summary, our findings not only reveal the mechanism by which hyperlactatemia mediates hepatic IRI but also identify PCK2 as a therapeutic target. Further clinical trials are necessary to investigate the beneficial effects of the rational utilization of PCK2 inhibitors on hepatic IRI injury in patients with altered serum lactate levels.

## 4 Experimental Section

### Human Samples

A retrospective analysis of all adults undergoing LT at West China Hospital of Sichuan University between May 2017 and August 2024 was performed. The patients were divided into two cohorts. Cohort 1 included 30 patients between May and August 2024, of whom 18 experienced hyperlactatemia during LT surgery and 12 did not. Lactate levels in venous blood were measured after donor liver implantation but before portal vein opening during LT surgery. The correlation between the serum ALT and AST levels of these 30 patients from POD1 to POD7 and lactate levels were assessed. Cohort 2 included 247 patients between May 2017 and April 2024. The clinical data of 100 patients from POD1 to POD3 were used to analyze the relationship between PCK2 expression levels and hepatic IRI through 1:1 PSM. Liver tissues were harvested before reperfusion (ischemia group) and after 1.5 h of reperfusion (reperfusion group). A portion of each liver sample was fixed in 4% formaldehyde and then embedded in paraffin, and the other portion was frozen directly in liquid nitrogen. All clinical data were obtained from records from the Chinese Liver Transplant Registry (CLTR, http://www.cltr.org/). Organs were donated voluntarily, and informed consent forms were signed by all involved donors or their families and by recipients. All samples were used only for scientific research, and the studies were performed according to the principles outlined in the Declaration of Helsinki. This study was approved by the Ethics Committee on Biomedical Research, West China Hospital of Sichuan University (2020, No 385).

### Experimental Animals

Eight‐week‐old male C57BL/6 mice were obtained from Byrness Weil Biotech Ltd. (Chengdu, China). All the mice were housed in a specific pathogen‐free (SPF) environment on a 12‐h light/dark cycle under controlled light, temperature, and humidity conditions. Food and water were available ad libitum. All operations on experimental animals were performed in accordance with the National Institutes of Health's Guide for the Care and Use of Laboratory Animals. All operations were approved by the Animal Care and Use Committee of West China Hospital of Sichuan University (2020338A).

### Animal Adeno‐Associated Virus (AAV) Production and Infection

To overexpress PCK2 in the liver, a hepatocyte‐specific AAV vector, AAV8‐TBG‐PCK2 (AAV‐PCK2) was constructed by GeneChem Co., Ltd. (Shanghai, China). An empty AAV8 vector (AAV‐V) was used as a negative control. Additionally, to knock down PCK2 or OXSM in the liver, AAV9‐TBG vectors harboring shRNAs against PCK2 or OXSM (AAV‐shPCK2 or shOXSM, designed and synthesized by GeneChem) were injected into the mice. Scramble shRNA (AAV‐sh) was used as a negative control. The TBG promoter was used to achieve hepatocyte‐specific overexpression or knockdown. For AAV transduction, viruses (1.5 × 10^11^ viral particles/mouse) were delivered via tail vein injection. After viral infection for 4 weeks, the mice were used for subsequent experiments. The AAV9‐TBG vectors harboring shRNAs were provided by Hanbio Biotechnology Co., Ltd. (Shanghai, China).

### Mouse Hepatic IRI Model

A mouse model of hepatic IRI was established as previously reported.^[^ ^33^ ^]^ In brief, after the mice were anesthetized with isoflurane, an upper abdominal midline laparotomy was performed to expose the liver. The blood supply to the left and median lobes was then occluded via an atraumatic clamp to induce ischemia for 90 min. Whitening of the ischemic liver lobe indicated successful ischemia induction. After the clamp was removed unless the indicated time points, liver samples, and serum were collected after 6 h of reperfusion for subsequent examination after reperfusion. Mice subjected to the same operation without vascular occlusion served as sham controls.

To investigate the roles of hyperlactatemia, ferroptosis and KAT8 inhibition in hepatic IRI, C57BL/6 mice was treated with lactate (120 mg k^−1^g, M10168, Abmole, Shanghai, China) in normal saline and Fer‐1 (20 mg k^−1^g, S7243, Selleck, Houston, TX), KAT8‐IN‐1 (10 mg k^−1^g, HY‐W015239, MCE, NJ, USA), or MC4033 (10 mg k^−1^g, HY‐149302, MCE) dissolved in 2% dimethyl sulfoxide (DMSO, D5879‐500ML, Sigma‒Aldrich). In accordance with the manufacturers’ instructions, lactate was intraperitoneally administered 1 h prior to ischemia induction, and Fer‐1, KAT8‐IN‐1, and MC4033 were intraperitoneally administered 2 h prior to ischemia induction. Mice in the control group were treated with the same volume of normal saline or DMSO.

### Mouse Liver Function Analysis

Whole blood samples were collected and centrifuged for 8 min at 4000 rpm to obtain serum. The serum levels of ALT and AST in the mice were measured via a biochemical analyzer (Sysmex) at the Clinical Test Analysis Department of Haiqi Pharmaceutical Technology Co., West China Hospital (Chengdu, China).

### Primary Hepatocyte Isolation and Culture

MPHs were isolated from the indicated male C57BL/6 mice via the type IV collagenase perfusion method as previously reported.^[^ ^33^ ^]^ In brief, the liver was digested by sequential perfusion of 50 mL of warm (37 °C) liver perfusion medium (PYG0081, Boster, Wuhan, China) and a solution containing 0.5% type IV collagenase (LS004188, Worthington, NJ, USA) via the portal vein. The liver was then excised, minced, and soaked in 0.5% type IV collagenase for further digestion; the digested material was filtered through a 70 µm cell strainer and washed with sterile PBS. MPHs in DMEM (Gibco, 11 885 084, NY, USA) supplemented with 10% fetal bovine serum (FBS, Gibco, 16 140 063, NY, USA) were seeded in plates coated with type I rat tail collagen (Merck, C3867, Shanghai, China) and cultured in an incubator containing 5% CO_2_ at 37 °C.

### Cell Culture

The THLE2 cell line was purchased from the Meisen Chinese Tissue Culture Collection (MeisenCTCC, Zhejiang, China). The HEK293T cell line was purchased from the Cell Bank of the Chinese Academy of Sciences (Shanghai, China). THLE2 cells were cultured in BEGM (MeisenCTCC), and HEK293T cells were cultured in high‐glucose DMEM (Gibco). The culture media were supplemented with 10% FBS and 1% penicillin‐streptomycin. The cells were cultured in a humidified incubator at 5% CO_2_ and 37 °C. All the cell lines were validated via STR DNA profiling and tested negative for mycoplasma via PCR.

### In Vitro H/R Model

To simulate hepatic IRI in vitro, H/R injury to MPH and THLE2 cells was induced. In brief, the cell medium was replaced with serum‐free medium, and the cells were cultured under hypoxic conditions (1% O_2_, 5% CO_2_, and 94% N_2_) for 4 h; then, the medium was replaced with complete medium, and the cells were transferred to a normoxic incubator for 2 h to allow reoxygenation. To investigate the role of lactate in H/R injury, MPHs, and THLE2 cells were treated with lactate (20 mM) for 0.5 h prior to hypoxia. Control cells were treated with normal saline.

### RNA Interference

Lipofectamine 3000 reagents (Invitrogen, Carlsbad, MA, USA) were used for siRNA transfection as described previously.^[^ ^18^, ^34^ ^]^ siRNAs (generated by Tsingke, Tianjin, China) were used at a final concentration of 50 nM. After transfection, the cells were cultured for an additional 48 h and then used in subsequent assays. Information about the siRNAs used in this study is provided in Table S5 (Supporting Information).

### Plasmid Construction and Transfection

PCR‐amplified human wild‐type PCK2, KAT8, and OXSM transcripts were cloned and inserted into the GV141‐Flag, GV141‐HA, and GV141‐Myc vectors, respectively. PCR‐amplified human wild‐type Parkin, MUL1, MARCH5, and RNF185 transcripts were cloned and inserted into the GV141‐HA vector. The ubiquitin (Ub) sequence was subsequently cloned and inserted into the GV141‐His vector. The site‐specific mutants used in this study were designed and synthesized by GeneChem (Shanghai, China). Additionally, to knock down PCK2 in THLE2 cells, shRNAs against PCK2 were designed and synthesized by GeneChem. Scramble shRNA was used as a negative control. All reagents used for the experiments described in this section were purchased from GeneChem. Information about the shRNAs used in this study is provided in Table S6 (Supporting Information). Transfection was performed with Lipofectamine 3000 reagent as described previously.^[^ ^18^, ^34^ ^]^

### CoIP

CoIP was performed via a Co‐IP kit (Abs955, Absin, Shanghai, China) as described previously.^[^ ^18^, ^34^ ^]^ In brief, hepatocytes were homogenized in IP lysis buffer (20 mM Tris–HCl (pH 7.5), 0.5% NP‐40, 250 mM NaCl, 3 mM EDTA, 3 mM EGTA, 1 mM DTT, 1 mM cocktail, 1 mM phosphoSTOP, 1 mM NEM, and 1 mM NAM). A total of 500 µg of the extract was incubated first with the indicated primary antibody or IgG as a negative control for 4 h and then with protein A/G‐Sepharose beads for 2 h at 4 °C. After extensive washes with PBS, the immunoprecipitates were used in subsequent assays. All the antibodies used were purchased from commercial manufacturers, as listed in Table S7 (Supporting Information).

### GST Pull‐Down Assay

GST‐fused PCK2 was expressed in *Escherichia coli* Rosetta cells and purified. Synthetic proteins were purified to >98% purity via high‐pressure liquid chromatography. HEK293T cell extracts expressing the indicated proteins were mixed with 5 mg of GST derivatives bound to glutathione‐Sepharose beads in 0.5 mL of modified binding buffer (25 mM Tris‐HCl at pH 7.2, 150 mM NaCl, 1 mM DTT, 0.5 mM EDTA, 0.5 mM EGTA, 1 mM cocktail, 1 mM phosphoSTOP, 1 mM NEM, and 1 mM NAM). The binding reaction was performed at 4 °C overnight, and the beads were subsequently washed 4 times with binding buffer and subjected to Western blot analysis.

### LC‐MS/MS Analysis

Immunoprecipitates of MPHs and THLE2 cells were resolved by SDS‒PAGE; the gel bands were digested with 10 ng µL^−1^ trypsin; and the proteins were eluted with 0.1% formic acid and 75% acetonitrile. The eluted proteins were then subjected to quality control and qualitatively analyzed via a Q ExactiveTM HF‐X mass spectrometer by Novogene Co., Ltd. (Tianjin, China) to obtain raw proteome data. The raw proteome data files were directly imported into Proteome Discoverer 2.2 software for database searching, peptide spectrum matching and protein quantification. Target protein‐protein interaction networks were constructed via the STRING database (https://string‐db.org/).

### Metabolomic Analysis

Metabolomic analyses were conducted by Bioprofile Technology Co., Ltd (Shanghai, China). In brief, after being frozen in liquid nitrogen, liver tissues were pulverized in 250 µL of mixed solvent (chloroform:methanol: water, 1:2:1). The lysate was sonicated and centrifuged for 10 min at 12000 rpm. The aqueous supernatant was transferred to a gas chromatography vial containing internal standards, and the pellet was resuspended in 250 µL of methanol and homogenized with a T10 basic homogenizer at 4 °C for 30 s. Aliquots of the supernatant and the mixture in the vial were vacuum‐dried after a second centrifugation. The samples were analyzed on an LC–MS/MS instrument (Thermo Fisher Ult3000‐Exploris 480 Orbitrap).

### RNA Sequencing

Total RNA was extracted from mouse livers via a TRIzol reagent kit (Takara, Dalian, China). Transcriptome sequencing and subsequent data analysis were conducted by Novogene Co., Ltd. (Tianjin, China). Bioinformatics analyses were performed as previously described.^[^ ^18^, ^34^ ^]^ In brief, the R “limma” package was used to identify differentially expressed genes between the indicated groups on the basis of the following criteria: false discovery rate (FDR) <0.05 and |log2(fold change (FC))|>1. The Database for Annotation, Visualization, and Integrated Discovery (DAVID) tool was used to analyze the enriched GO and KEGG pathways. GSEA was performed to analyze the GO categories in which the signature gene sets from the gene profiles between the indicated groups were enriched.

### TEM

Fresh liver tissues of appropriate size were obtained and immediately fixed in 2.5% phosphate‐glutaraldehyde for 4 h. After 2 washes in dimethylarsenic acid sodium buffer, the samples were dehydrated in an ethanol gradient and then fixed, embedded, and sectioned (70 nm). The sections were then viewed with a Tecnai 10 (100 kV) transmission electron microscope. For each sample, five fields of view were randomly selected, and twenty mitochondria were examined in each field of view.

### Histology and IHC

Histology and IHC were performed as previously described.^[^ ^18^, ^34^ ^]^ In brief, paraffin‐embedded liver tissues were serially sectioned at a thickness of 5 µm. The sections were then stained with HE for routine histological examination via light microscopy. To measure the iron content, selected sections were stained with Prussian blue. In addition, serial tissue sections were deparaffinized, subjected to antigen retrieval using 0.01 m citric acid buffer (pH 6.0) at 95 °C for 15 min, and incubated overnight at 4 °C with primary antibodies prior to incubation with HRP‐conjugated AffiniPure goat anti‐rabbit IgG (Proteintech, SA00001‐2, Wuhan, China; 1:5000) for 1 h at room temperature. Information about the primary antibodies used in this study is available in Table S7 (Supporting Information).

### TUNEL Assay

Liver tissue sections and formalin‐fixed paraffin‐embedded cells were deparaffinized, and TUNEL (BOSTER, MK1028, Wuhan, China) staining was performed according to the manufacturer's instructions.

### Calcein‐AM/PI Staining

To analyze the death of hepatocytes directly, calcein‐AM/PI staining was performed via a Calcein/PI Cell Viability/Cytotoxicity Assay Kit (Beyotime, C2015S, Wuhan, China) in accordance with the manufacturer's instructions. Five fields of view for each sample were randomly photographed (Zeiss, Oberkochen, Germany), and the average value for the five fields of view is reported for each sample. The cell death ratio was calculated as the number of PI‐positive cells/the number of total cells.

### Lipid Peroxidation Analysis

The levels of ROS, MDA, GSH, and SOD were measured via commercially available kits from Nanjing Jiancheng Bioengineering Institute (Nanjing, China) in accordance with the manufacturer's instructions. In brief, liver tissues or cells were rinsed with PBS, homogenized in RIPA lysis buffer on ice, and sonicated. After sonication, the samples were centrifuged at 4000 rpm for 10 min to remove debris. The protein concentration of each sample was determined with a BCA protein assay kit (P0009, Beyotime). Using a microplate reader (Thermo, Multiskan SkyHigh, A51119500C), the ROS level (E004‐1‐1) was measured on the basis of the content of 2,7‐dichlorofuorescin diacetate detected at 488 nm; the MDA level (A003‐1‐2) was measured on the basis of the content of thiobarbituric acid‐reactive substances detected at 532 nm; the GSH level (A006‐2‐1) was measured on the basis of the content of 2‐nitrobenzoic acid‐reactive substances detected at 412 nm; and the SOD level (A001‐3‐2) was measured on the basis of the content of WST1‐reactive substances detected at 450 nm. Finally, ROS, MDA, GSH, and SOD levels were normalized to the protein concentration for statistical analysis.

### Measurement of Iron Content

Total elemental iron was measured via a tissue iron assay kit (A039‐2‐1, Jiancheng) according to the manufacturer's instructions. The iron content was normalized to the protein concentration for statistical analysis.

### Measurement of PEPCK Activity

Intracellular PEPCK activity was measured via a phosphoenolpyruvate carboxylase test kit (A130‐1‐1, Jiancheng) according to the manufacturer's protocol. PEPCK activity was normalized to the protein concentration for statistical analysis.

### Seahorse Assay

The indicated THLE2 cells were plated in an XF96 plate (W21715, Seahorse Biosciences, USA) at a density of ≈1 × 10^4^ cells/well. Oxygen consumption was measured with a Seahorse XFe96 Analyzer (Agilent) to analyze the maximum OXPHOS respiration capacity. After basal readings were obtained, 1.5 mM oligomycin, 0.5 mM FCCP, and 0.5 mM rotenone/antimycin A were injected in sequence. All drugs were provided in the Cell Mito Stress Test Kit for XFe/XF Analyzers (Agilent, 103015–100). The glycolytic rate was also measured with a Seahorse XFe96 Analyzer. After basal readings were obtained, 0.5 mM rotenone/AA and 50 mM 2‐deoxy‐D‐glucose (2‐DG) were injected in sequence. All drugs were provided in the Seahorse XF Glycolytic Rate Assay Kit (Agilent, 103344‐100).

### qRT‒PCR

Total RNA was isolated from tissues and cells via TRIzol (Thermo Fisher Scientific, 15 596 018, WI, USA). The RNA concentration and purity were measured via a spectrophotometer. The RNA was reverse transcribed via the PrimeScript RT Reagent Kit (Takara), and qPCR was performed via SYBR Premix Ex Taq (Takara) according to the manufacturer's protocol. The expression levels were normalized to the endogenous expression level of β‐actin, which was used as the control. The thermocycling conditions were as follows: initial denaturation at 95 °C for 30 s, followed by 60 cycles of denaturation at 95 °C for 5 s, annealing at 55 °C for 30 s, and extension at 72 °C for 30 s. Information about the primers used in this study is available in Table S8 (Supporting Information).

### Western Blot Analysis

Protein was extracted from liver tissues and hepatocytes via RIPA buffer (Sigma‒Aldrich, Darmstadt, Germany) containing 1% phenylmethylsulfonyl fluoride (PMSF) (Beyotime, ST506, Shanghai, China) and phosphatase inhibitors (Beyotime, P1045). The protein concentration of each sample was determined with a BCA protein assay kit (Beyotime, P0009). Denatured protein samples (10 µg) were separated via 8% SDS‒PAGE (Zoman, Beijing, China) and transferred onto nitrocellulose (NC) membranes. The NC membranes were blocked with 5% nonfat dried milk in Tris‐buffered saline (pH 7.4) containing 0.1% Tween 20 (TBST) for 1 h at 4 °C and subsequently reacted with specific primary antibodies diluted in TBST at 4 °C overnight. All primary antibodies were used at a dilution of 1:1000. The membranes were subsequently washed three times in TBST for 10 min each and incubated with an HRP‐conjugated AffiniPure goat anti‐rabbit IgG (H + L) (Proteintech, SA00001‐2, Wuhan, China; 1:4000) secondary antibody for 1 h at room temperature. The signals were detected via enhanced chemiluminescence (Meilunbio, Dalian, China). An anti‐β‐actin antibody was used for the normalization of protein expression. For cycloheximide (CHX) chase assays, hepatocytes were treated with 20 mg mL^−1^ CHX for 24 h after transfection and collected at the indicated time points, and the resulting cell lysates were subjected to Western blotting. Information about the primary antibodies used in this study is available in Table S7 (Supporting Information).

### Molecular Docking Analysis

The structure of KAT8 (AFDB accession: AF‐Q9H7Z6‐F1‐v4) was downloaded from the AlphaFold Protein Structure Database. The docking of KAT8 with lactyl coenzyme A (lactyl‐CoA) or acetyl coenzyme A (acetyl‐CoA) was assessed via the GRAMM Docking Web Server (https://gramm.compbio.ku.edu) and further analyzed via Discovery Studio software according to previously reported methods.^[^ ^35^ ^]^

### Statistical Analysis

All the statistical analyses in this study were performed via SPSS 22.0 (IBM Corp.), and the figures were produced via GraphPad Prism 6.0 or R software (version 3.6.1). All the experiments were independently performed at least three times, and n represents the number of independent samples or mice from each subgroup. The data are expressed as the means ± standard deviations (SDs). The normal distribution of the data was assessed by the Kolmogorov‐Smirnov test. For normally distributed data, the data were analyzed via unpaired Student's t‐test or one‐way ANOVA followed by Bonferroni's post hoc test, where appropriate. For non‐normally distributed data, Kruskal–Wallis tests were used to compare differences among more than two groups, and Wilcoxon tests to compare differences between two groups. For analysis of the clinical data, the correlation between two parameters was evaluated via Pearson correlation analysis. Statistical significance was evaluated on the basis of *p* values, and *p* < 0.05 was considered to indicate statistical significance.

## Conflict of Interest

The authors declare no conflict of interest.

## Author Contributions

Y. S., J. Y., and J. Y. designed the project. J. Y., M. Y., Z. W., J. W., K. Z., J. W., Q. Z., M. C., and T. L. performed the experiments and acquired the data. J. Y. and J. Y. analyzed the results and wrote the manuscript.

## Supporting information



Supporting Information

Supplementary Table1

## Data Availability

The data that support the findings of this study are available from the corresponding author upon reasonable request.
