# Supplementary material for: The Lactate‐Primed KAT8‒PCK2 Axis Exacerbates Hepatic Ferroptosis During Ischemia/Reperfusion Injury by Reprogramming OXSM‐Dependent Mitochondrial Fatty Acid Synthesis
Source: Adv Sci (Weinh). 2025 Jan 24;12(11):2414141. doi: 10.1002/advs.202414141 (PMC11923996; doi:10.1002/advs.202414141)
Supplement: Supplementary file 1 — Supporting Information [file ADVS-12-2414141-s002.pdf]

ADVANCED SCIENCE
Open Access

Supporting Information

for *Adv. Sci.*, DOI 10.1002/advs.202414141

The Lactate-Primed KAT8–PCK2 Axis Exacerbates Hepatic Ferroptosis During Ischemia/Reperfusion Injury by Reprogramming OXSM-Dependent Mitochondrial Fatty Acid Synthesis

Jingsheng Yuan, Mingyang Yang, Zhenru Wu, Jun Wu, Kejie Zheng, JiaGuo Wang, Qiwen Zeng, Menglin Chen, Tao Lv, Yujun Shi, Jiayin Yang* and Jian Yang**

Supporting Information

The Lactate-Primed KAT8–PCK2 Axis Exacerbates Hepatic Ferroptosis During Ischemia/Reperfusion Injury by Reprogramming OXSM-Dependent Mitochondrial Fatty Acid Synthesis

Jingsheng Yuan, Mingyang Yang, Zhenru Wu, Jun Wu, Kejie Zheng, JiaGuo Wang, Qiwen Zeng, Menglin Chen, Tao Lv, Yujun Shi, Jiayin Yang*, Jian Yang**

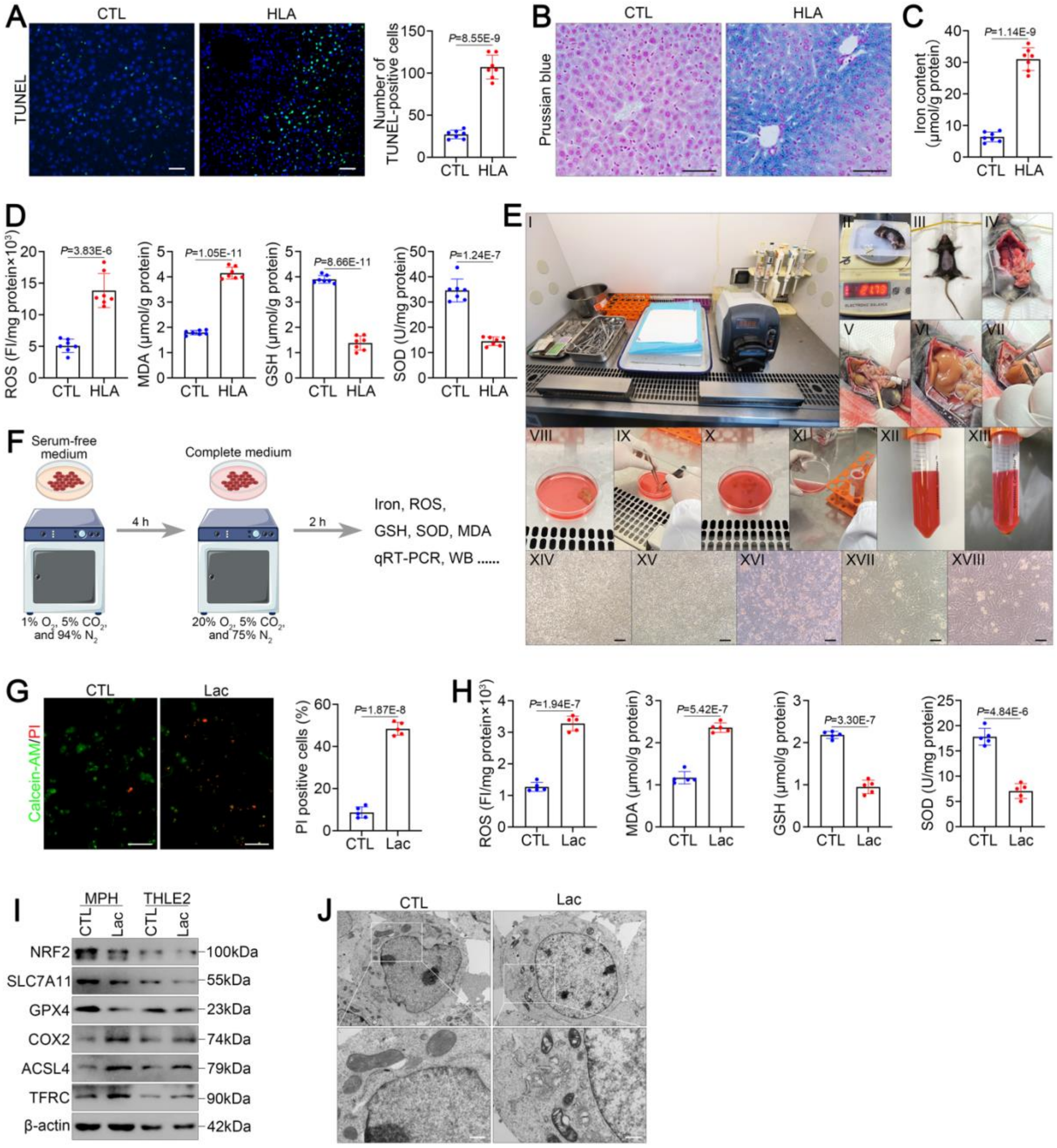

Figure S1. Lactate is positively associated with ferroptosis during hepatic IRI. (A) Representative images and relative quantification of TUNEL staining in liver tissues from mice treated with/without lactate following IRI (scale bar =200 μm). (B) Representative images of Prussian blue staining of liver tissues from mice treated with/without lactate following IRI (scale bar =50 μm). (C) Iron content in liver tissues from mice treated with/without lactate following IRI. (D) ROS, MDA, GSH and SOD levels in liver tissues from mice treated with/without lactate following IRI. (E) I. Material preparation; II. Weighing; III. Skin preparation; IV. Open the abdomen and prepare the vena cava and portal vein; V. Cannulate the Vena cava caudally to the hepatic vein with a 25G butterfly needle; VI. Start the pump with a speed setting at 30 ~10 ml/min. After 1-2 seconds, liver will stand up,

then cut the portal vein. The liver should be free of blood within 30 s-1 min; VII. Remove the liver; VIII. Use cotton swaps to pick up liver to prewarmed 15 ml Wash buffer. IX. Liver is gently teased apart using two tweezers; X. Hepatocytes fall off, and not much tissue left-over; XI. Filter the cell suspension using a cell strainer; XII. Pool cell suspensions together in a 50 ml blue cap; XIII. Centrifuge (450rpm/3 minutes/4°C); XIV. Plate cells and culture cells at 37°C/5 % CO_2 (scale bar =200 μm); XV. Microscopic observation of hepatocytes after 24h of culture (scale bar =200 μm); XVI. Microscopic observation of hepatocytes after 48h of culture (scale bar =100 μm); XVII. Microscopic observation of hepatocytes after 72h of culture (scale bar =100 μm); XVIII. Microscopic observation of hepatocytes after 96h of culture (scale bar =100 μm). (F) Schematic diagram of cellular H/R damage model construction. (G) Representative images and relative quantification of calcein-AM/PI double-staining in THLE2 cells treated with/without lactate following H/R (scale bar =100 μm). (H) ROS, MDA, GSH and SOD levels in THLE2 cells treated with/without lactate following H/R. (I) Western blot analysis of ferroptosis-associated proteins in MPH and THLE2 cells treated with/without lactate following H/R. (J) Representative images of TEM in THLE2 cells treated with/without lactate following H/R (scale bar =500 nm). For all the above experiments, the data are presented as the means ± SDs. For G-J, three independent experiments (n = 3) with similar results were performed in triplicate. In A, C, D, G and H, the statistical analyses were performed via two-tailed unpaired Student's t-tests. $P < 0.05$ was considered statistically significant.

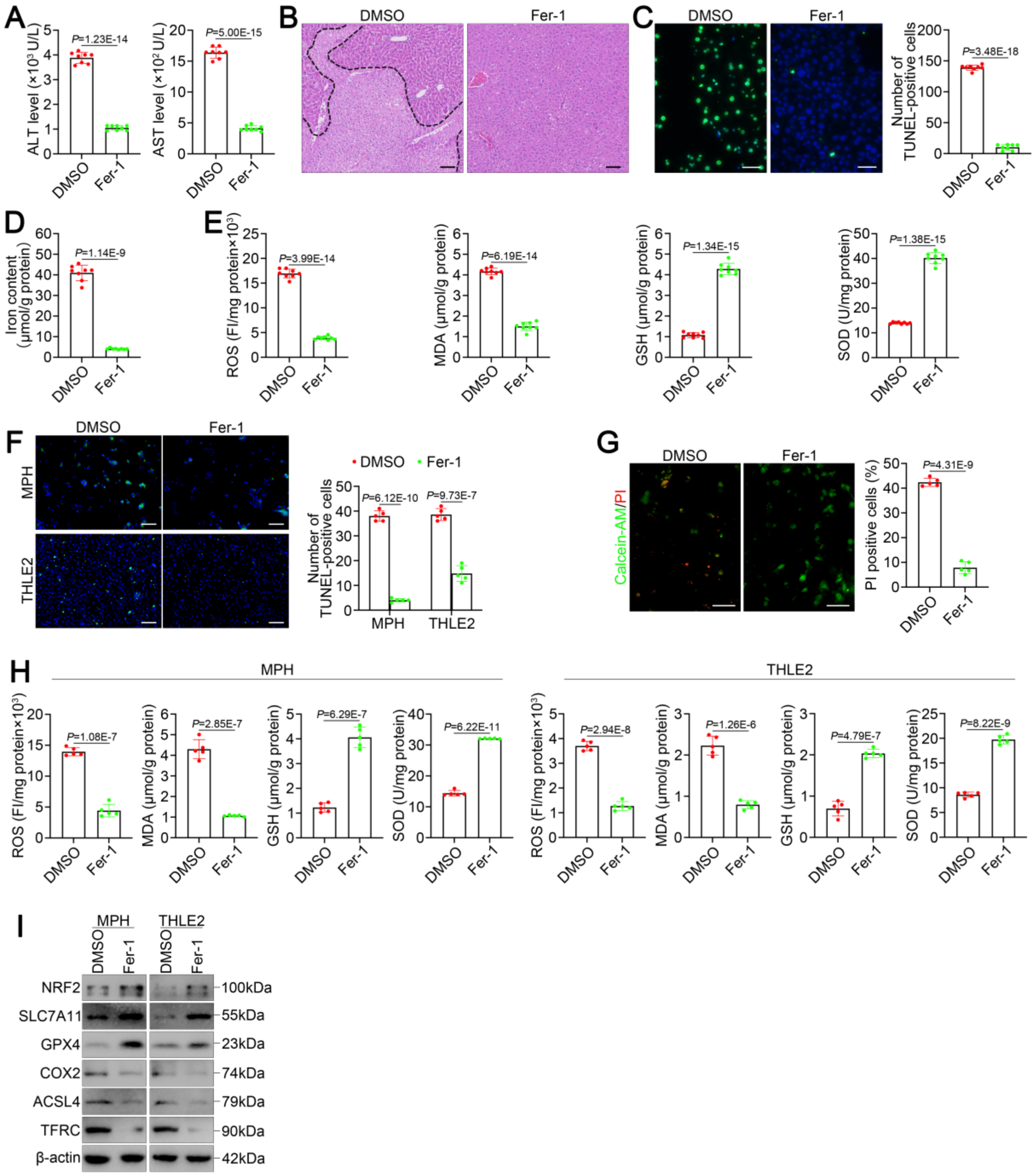

Figure S2. Fer-1 attenuates hyperlactatemia-mediated hepatic ferroptosis and IRI in mice. (A) Serum AST and ALT levels in mice (n = 8/group) treated with lactate and with/without Fer-1 following IRI. (B) Representative images of HE staining of liver tissues from mice treated lactate and with/without Fer-1 following IRI (scale bar =100 μm). (C) Representative images and relative quantification of TUNEL staining in liver tissues from mice treated with lactate and with/without Fer-1 following IRI (scale bar =100 μm). (D) Iron content in liver tissues from mice treated with lactate and with/without Fer-1 following IRI. (E) ROS, MDA, GSH and SOD levels in liver tissues from mice treated with lactate and with/without Fer-1

following IRI. (F) Representative images and relative quantification of calcein-AM/PI double-staining in MPH and THLE2 cells treated with lactate and with/without Fer-1 following H/R (scale bar =200 μm). (G) Representative images and relative quantification of TUNEL staining in MPH and THLE2 cells treated with lactate and with/without Fer-1 following H/R (scale bar =100 μm). (H) ROS, MDA, GSH and SOD levels in MPH and THLE2 cells treated with lactate and with/without Fer-1 following H/R. (I) Western blot analysis of ferroptosis-associated proteins in MPH and THLE2 cells treated with lactate and with/without Fer-1 following H/R. For all the above experiments, the data are presented as the means ± SDs. For F-I, 3 independent experiments (n = 3) with similar results were performed in triplicate. In A and C-H, the statistical analyses were performed by two-tailed unpaired Student's t-test. $P < 0.05$ was considered statistically significant.

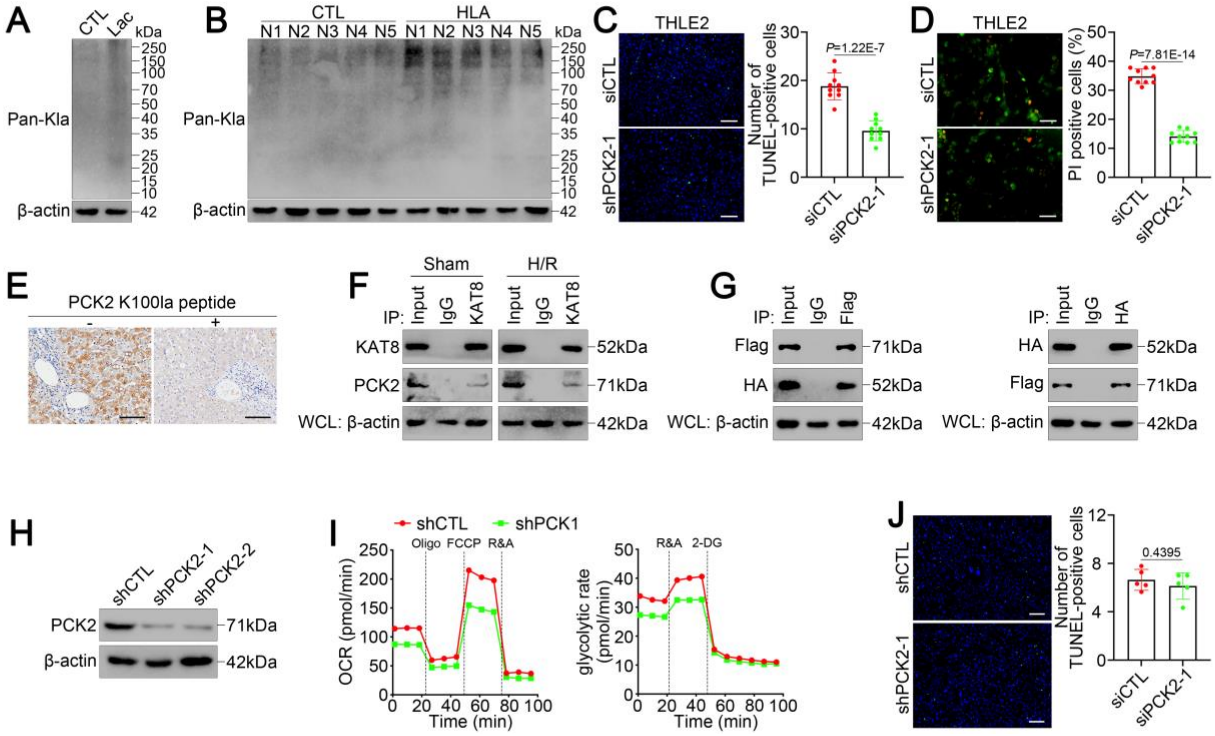

Figure S3. KAT8 lactylates PCK2-K100 in high-lactate-cultured hepatocytes. (A) Western blot analysis of pan-Kla of lysates from MPH cells treated with/without lactate following H/R. (B) Western blot analysis of pan-Kla of liver tissues from mice treated with/without lactate following IRI. (C) Representative images and relative quantification of TUNEL staining in THLE2 cell cultured with/without lactate and transfected with/without shPCK2 and subjected to H/R, as indicated (Scale bar = 200 μm). (D) Representative images and relative quantification of calcein-AM/PI double-staining in THLE2 cell cultured with/without lactate and transfected with/without shPCK2 and subjected to H/R, as indicated (Scale bar = 100 μm). (E) PCK2 K100 lactylation-specific antibody was validated for IHC staining. Representative images showing liver tissues stained with the antibody of PCK2K100lac incubated in the presence or absence of lactylated PCK2 peptides (Scale bar = 100 μm). (F) THLE2 cell lysates before and after reperfusion were incubated with an anti-KAT8 antibody, and interacting proteins were detected with an anti-PCK2 antibody using western blotting as indicated. (G) THLE2 cell lysates were incubated with an anti-Flag or anti-HA antibody, and interacting proteins were detected with an anti-HA or anti-Flag antibody using western blotting as indicated. (H) Western blot analysis of PCK2 in THLE2 cells transfected with/without shPCK2. (I) Real-time seahorse OCR and glycolytic rate measurements for OXPHOS and TCA cycle speed evaluation in THLE2 cells transfected with/without shPCK2. (J) Representative images and relative quantification of TUNEL staining in THLE2 cell cultured with/without lactate and transfected with/without shPCK2 (Scale bar = 200 μm). For

all the above experiments, the data are presented as the means ± SDs. Three independent experiments (n = 3) with similar results were performed in triplicate. In C, D and J, the statistical analyses were performed by two-tailed unpaired Student's t-test. $P < 0.05$ was considered statistically significant.

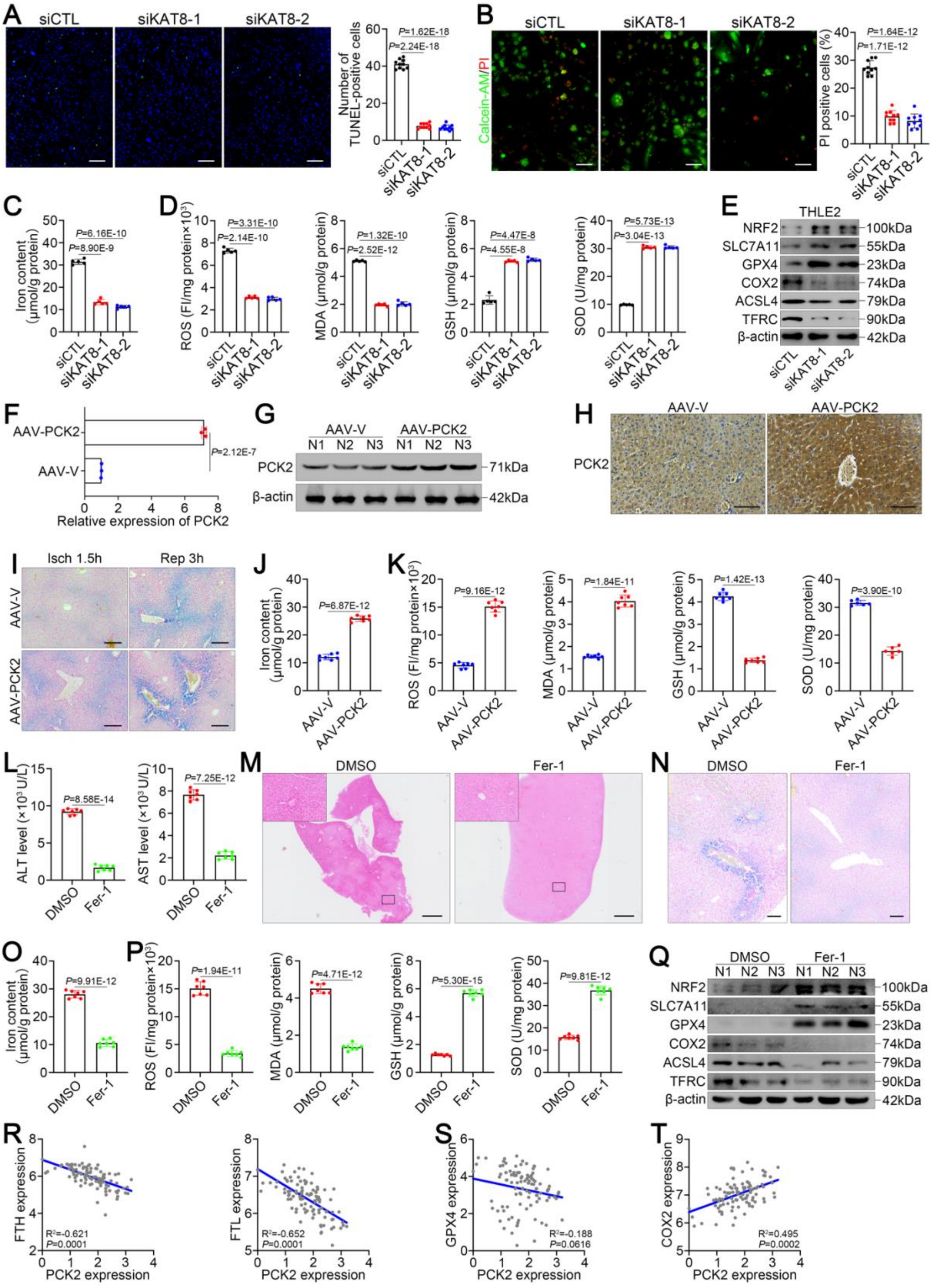

Figure S4. PCK2 renders livers susceptible to ferroptosis and IRI. (A-E) THLE2 cells were transfected with siCTL or siKAT8 and subjected to H/R (A) Representative images and relative quantification of TUNEL staining (scale bar =200 μm). (B) Representative images

and relative quantification of calcein-AM/PI double-staining (scale bar =50 μm). (C) Iron content. (D) ROS, MDA, GSH and SOD levels. (E) Western blot analysis of ferroptosis-associated proteins. (F-K) Hepatic IRI in AAV-V or AAV-PCK2 mice (n = 7/group). (F) qRT–PCR analysis of PCK2 in AAV-PCK2 and AAV-V livers. (G) Western blot analysis of PCK2 in AAV-PCK2 and AAV-V livers. (H) Representative images of IHC staining of PCK2 in AAV-PCK2 and AAV-V livers. (scale bar =100 μm). (I) Representative images of Prussian blue staining of liver tissues from AAV-PCK2 and AAV-V mice subjected to IRI (scale bar =200 μm). (J) Iron content of liver tissues from AAV-PCK2 and AAV-V mice subjected to IRI. (K) ROS, MDA, GSH and SOD levels of liver tissues from AAV-PCK2 and AAV-V mice subjected to IRI. (L-Q) AAV-V or AAV-PCK2 mice were treated with/without Fer-1 and subjected to IRI (n = 7/group). (L) Serum AST and ALT levels. (M) Representative images of HE-stained livers (scale bar =1 mm). (N) Representative images of Prussian blue staining of livers (scale bar =100 μm). (O) Iron content of liver tissues. (P) ROS, MDA, GSH and SOD levels of liver tissues. (Q) Western blot analysis of ferroptosis-associated proteins. (R-T) Pearson correlation analysis between PCK2 and ferroptosis-associated proteins in donor livers in cohort 2 at POD1 (n = 100). For all the above experiments, the data are presented as the means ±SDs. Three independent experiments (n = 3) with similar results were performed in triplicate. In A-D, F, J-L and O-I, the statistical analyses were performed by two-tailed unpaired Student's t-test, respectively. $P < 0.05$ was considered statistically significant.

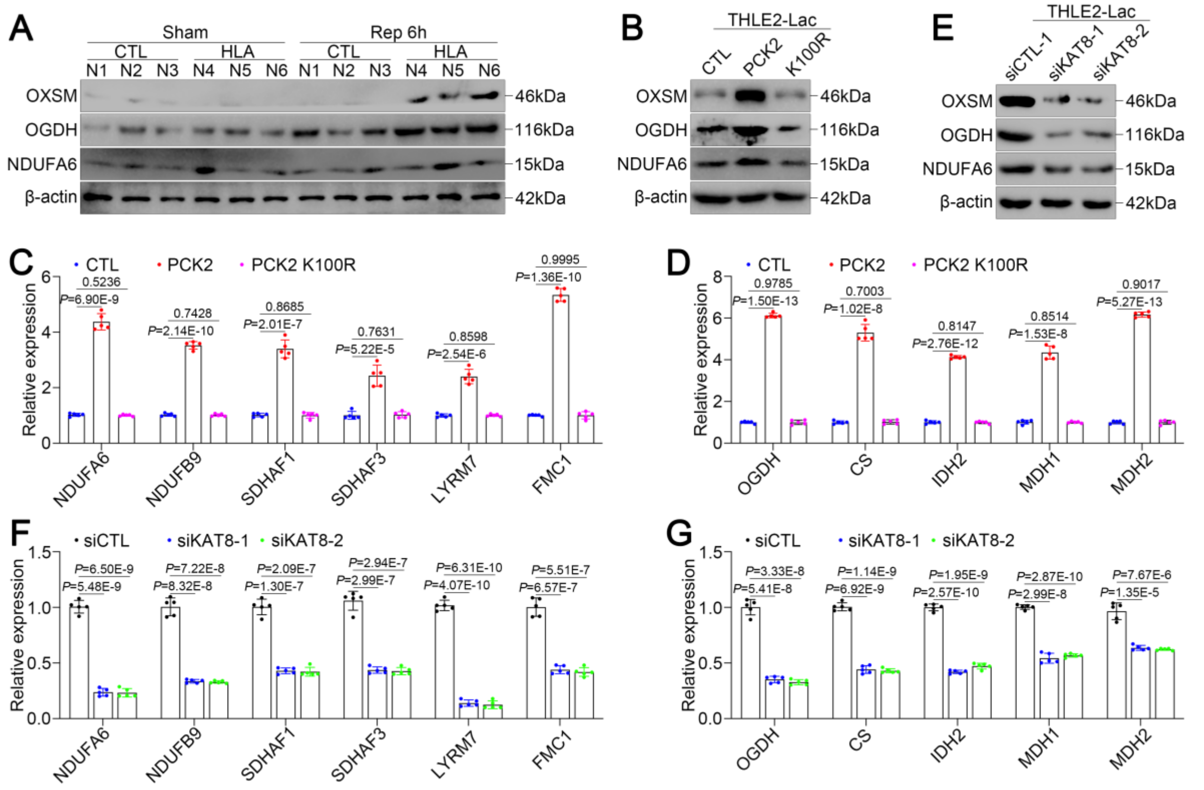

Figure S5. Hyperlactatemia facilitates mtFAS metabolic reprogramming. (A) Western blot analysis of OXSM, OGDH and NDUFA6 in liver tissues from mice treated with/without lactate and subjected to IRI. (B) Western blot analysis of OXSM, OGDH and NDUFA6 in high-lactate-cultured PCK2^{KD} THLE2 cells transfected with Flag-CTL, Flag-PCK2 or Flag-PCK2 K100R and subjected to H/R. (C-D) qRT–PCR analysis of the top significantly downregulated (C) OXPHOS genes and (D) TCA cycle genes in high-lactate-cultured PCK2^{KD} THLE2 cells transfected with Flag-CTL, Flag-PCK2 or Flag-PCK2 K100R and subjected to H/R. (E) Western blot analysis of OXSM, OGDH and NDUFA6 in high-lactate-cultured THLE2 cells transfected with siCTL or siKAT8 and subjected to H/R. (F-G) qRT–PCR analysis of the top significantly downregulated (F) OXPHOS genes and (G) TCA cycle genes in high-lactate-cultured THLE2 cells transfected with siCTL or siKAT8 and subjected to H/R. For all the above experiments, the data are presented as the means ± SDs. Three independent experiments (n = 3) with similar results were performed in triplicate. In C-D and F-G, the statistical analyses were performed by two-tailed unpaired Student's t-test. $P < 0.05$ was considered statistically significant.

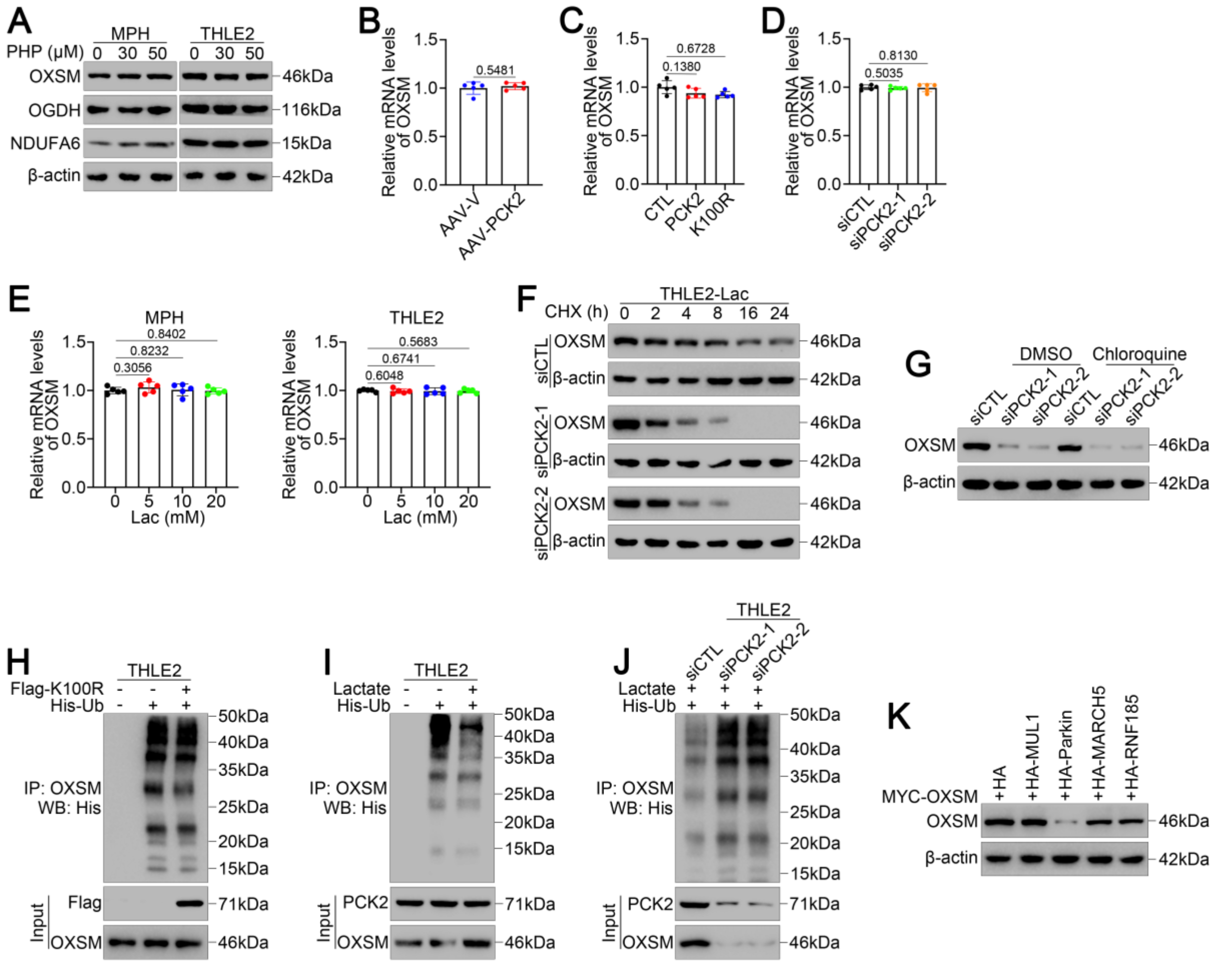

Figure S6. PCK2 inhibits OXSM ubiquitination degradation. (A) Western blot analysis of OXSM, OGDH and NDUFA6 in MPH and THLE2 cells treated with indicated dose of phosphoenolpyruvate (PHP). (B) qRT–PCR analysis of OXSM in AAV-PCK2 and AAV-V livers. (C) qRT–PCR analysis of OXSM in THLE2 cells transfected with Flag-CTL, Flag-PCK2 or Flag-PCK2 K100R and subjected to H/R. (D) qRT–PCR analysis of OXSM in THLE2 cells transfected with siCTL or siPCK2 and subjected to H/R. (E) qRT–PCR analysis of OXSM in MPH and THLE2 cells treated with indicated dose of lactate (Lac). (F) Western blot analysis of OXSM in high-lactate-cultured THLE2 cells transfected with siCTL or siPCK2 and treated with CHX for the indicated times. (G) Western blot analysis of OXSM protein expression in the indicated Flag-PCK2-overexpressing THLE2 cells transfected with siCTL or siPCK2 and treated with/without chloroquine as indicated. (H-J) THLE2 cells were transfected as indicated, and the cell lysates were then immunoprecipitated with an anti-OXSM antibody and detected with an anti-His antibody. (K) Western blot analysis of OXSM protein expression in MYC-OXSM-overexpressing THLE2 cells transfected with indicated overexpressing vectors. For all the above experiments, the data are presented as the means ± SDs. Three independent experiments (n = 3) with similar results were performed in triplicate.

In B-E, the statistical analyses were performed by two-tailed unpaired Student's t-test. $P < 0.05$ was considered statistically significant.

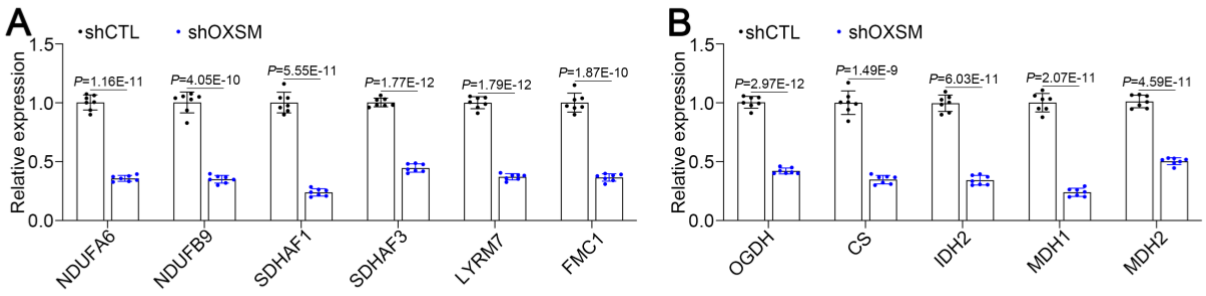

Figure S7. PCK2-mediated OXSM-dependent ferroptosis during hepatic IRI. AAV-PCK2 mice infected with AAV-shCTL or AAV-shOXSM were treated with lactate and subjected to hepatic IRI. qRT–PCR analysis of the top significantly downregulated (A) OXPHOS genes and (B) TCA cycle genes in the liver. For all the above experiments, the data are presented as the means ± SDs. The statistical analyses were performed by two-tailed unpaired Student's t-test. $P < 0.05$ was considered statistically significant.

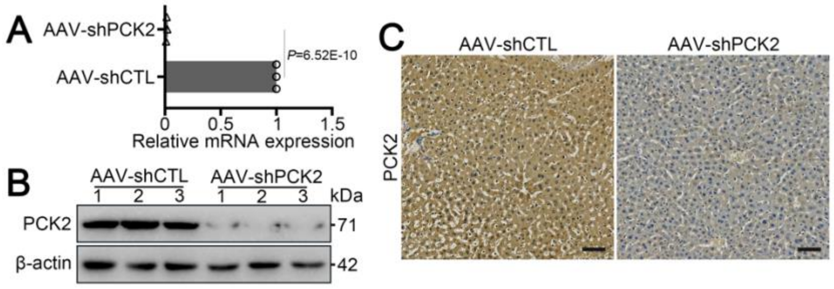

Figure S8. Targeting PCK2 attenuates hyperlactatemia-mediated hepatic ferroptosis during IRI. (A) qRT–PCR analysis of PCK2 in AAV-shPCK2 and AAV-shCTL livers. (B) Western blot analysis of PCK2 in AAV-shPCK2 and AAV-shCTL livers. (C) Representative images of IHC staining of PCK2 in AAV-shPCK2 and AAV-shCTL livers (scale bar =100 μm). For A, the data are presented as the means ± SDs, the statistical analyses were performed by two-tailed unpaired Student's t-test, $P < 0.05$ was considered statistically significant. For A-B, three independent experiments (n = 3) with similar results were performed in triplicate.

Table S1. The lysine-lactylated substrates in THLE2 cells treated with/without lactate following H/R.

Table S2. The PCK2-binding protein library from THLE2 cell lysates.

Table S3. Baseline characteristics of adult liver transplantation donors and recipients based on Pck2 expression.

Variables Donors		High (n=50)	Low (n=50)	P
Age (years)		48.140±11.997	45.020±14.056	0.235
Sex	Male	34	35	0.829
	Female	16	15	
Blood type	O	21	19	0.774
	A	17	15	
	B	7	11	
	AB	5	5	
Weight (Kg)		60.700±11.601	64.330±9.784	0.094
High (cm)		163.600±9.214	166.660±6.962	0.064
BMI (Kg/m^2)		22.492±3.319	23.076±2.572	0.328
HGB (g/L)		108.939±30.592	106.592±32.586	0.711
WBC (10^9/L)		13.007±7.096	12.676±5.781	0.799
PLT (10^9/L)		119.388±63.420	124.735±62.520	0.672
Na^+ (mmol/L)		153.465±12.159	153.469±11.248	0.999
K^+ (mmol/L)		3.990±0.638	4.068±1.057	0.654
UREA (mmol/L)		7.993±5.478	9.676±6.639	0.170
CREA (μmol/L)		134.967±161.096	129.326±114.838	0.841
ALB (g/L)		36.161±8.647	35.588±7.441	0.723
TB (μmol/L)		16.638±11.835	16.637±12.344	0.999
ALP (IU/L)		77.667±30.717	91.334±45.732	0.083
γGT (IU/L)		38.413±46.644	52.768±55.507	0.165
AST (IU/L)		65.263±90.734	50.386±53.514	0.320
ALT (IU/L)		48.651±59.642	43.158±40.076	0.590
GLU (mmol/L)		9.819±4.997	9.953±6.005	0.904
Graft weight (g)		1142.167±425.322	1253.180±427.002	0.196
Warm IS time (min)		24.830±7.473	24.000±8.238	0.599
Perfusion time (min)		14.620±5.591	16.420±5.380	0.104
Variables recipients				
Sex	Male	34	36	0.663
	Female	16	14	
Age (years)		51.660±9.958	47.320±11.313	0.044
High (cm)		155.300±28.203	164.420±16.839	0.052
Weight (Kg)		57.336±17.612	61.370±14.431	0.213
BMI (Kg/m^2)		22.690±2.905	22.268±3.471	0.512
CREA (μmol/L)		75.214±31.454	86.820±78.067	0.332
ALB (g/L)		35.956±7.475	36.186±6.611	0.871
TB (μmol/L)		195.926±198.196	190.632±215.822	0.906
INR		2.187±2.227	1.968±1.667	0.581
MELD		21.660±11.830	20.340±11.755	0.577
CHILD		9.580±1.864	9.420±2.278	0.702

Anhepatic stage (min)	92.560±33.454	86.680±40.496	0.431
Cold IS time (h)	390.520±144.537	385.180±155.195	0.859
Total operation time (h)	510.140±117.461	486.120±119.391	0.313

High: High expression, Low: Low expression, INR: international normalized ratio; MELD: model end-stage liver disease; IS: ischemia; HGB: hemoglobin; WBC: white blood cells; PLT: platelets; Na^+, serum sodium; K^+: serum potassium; CREA: creatinine; ALB: albumin; TBIL: total bilirubin; ALP: alkaline phosphatase; γGT: γ-glutamyl transpeptidase; GLU: blood glucose; AST: aspartate aminotransferase; ALT: alanine aminotransferase.

Table S4. Univariate analysis of factors associated with PCK2 after liver transplantation in recipients.

Variables recipients	High (n=50)	Low (n=50)	P
FIB (g/L)	2.300±0.936	2.119±0.926	0.333
APTTP (s)	43.605±17.577	45.802±17.018	0.527
INR	2.027±3.083	1.556±0.587	0.291
PT (s)	18.244±7.633	17.473±6.176	0.580
CREA (μmol/L)	78.897±42.091	79.863±42.562	0.909
UREA (mmol/L)	9.437±5.049	9.611±4.784	0.860
MG (mmol/L)	0.804±0.122	0.778±0.084	0.211
CA (mmol/L)	2.079±0.115	2.129±0.136	0.049*
K^+ (mmol/L)	4.078±0.501	4.087±0.446	0.920
Na^+ (mmol/L)	140.457±3.784	139.770±3.429	0.344
AMON (μmol/L)	66.270±50.706	68.997±50.316	0.788
GLU (mmol/L)	8.790±1.554	8.068±1.578	0.023*
CHOL (mmol/L)	2.609±1.090	2.530±0.824	0.683
TG (mmol/L)	1.334±0.531	1.222±0.580	0.314
LDH (IU/L)	838.697±2084.347	321.890±175.747	0.084
ALT (IU/L)	398.967±408.452	159.343±74.351	<0.001*
AST (IU/L)	593.860±1489.928	159.610±86.464	0.042*
γGT (IU/L)	132.689±96.151	141.947±161.489	0.728
ALP (IU/L)	173.527±103.909	154.757±134.819	0.437
DB (μmol/L)	78.278±88.022	84.683±88.413	0.717
TB (μmol/L)	95.768±98.397	111.850±111.555	0.446
ALB (g/L)	36.107±4.121	35.177±4.173	0.265
PLT (10^9/L)	70.163±46.091	67.047±57.782	0.766
WBC (10^9/L)	9.359±4.152	7.859±3.758	0.061
HGB (g/L)	98.387±16.085	97.080±16.842	0.692

FIB: fibrinogen; APTTP: activated partial thromboplastin time; INR: international normalized ratio; PT: prothrombin time; CREA: creatinine; MG: serum magnesium; CA: serum calcium; K^+: serum potassium; Na^+: serum sodium; AMON: blood ammonia; GLU: blood glucose; CHOL: cholesterol; TG: triglyceride; LDH: lactate dehydrogenase; AST: aspartate aminotransferase; ALT: alanine aminotransferase; ALP: alkaline phosphatase; γGT: γ-glutamyl transpeptidase; DB: direct bilirubin; TB: total bilirubin; ALB: albumin; PLT: platelets; WBC: white blood cells; HGB: Hemoglobin.

Table S5. Sequences of the siRNAs used in the study.

Primer names	Sequences (5'-3')
siPCK2-1 sense	GGUUGGUCUCAAACUCCUAAC
siPCK2-1 anti-sense	UAGGAGUUUGAGACCAACCUG
siPCK2-2 sense	CGUUGAUUAAACAGUUAAACA
siPCK2-2 anti-sense	UUUAACUGUUUAAUCAACGUU
siKAT8-1 sense	GGAAUAACUUGCUCAGUUACA
siKAT8-1 anti-sense	UAACUGAGCAAGUUAUUCCAU
siKAT8-2 sense	GGAAUUAUACAUUAAUGUAGG
siKAT8-2 anti-sense	UACAUUAAUGUAUAAUUCCAG
siOXSM-1 sense	GAGAGAUUCUGUAGUUCUAAC
siOXSM-1 anti-sense	UAGAACUACAGAAUCUCUCAG
siOXSM-2 sense	GGUCAGCAUUCGAUAUAAACU
siOXSM-2 anti-sense	UUUAUAUCGAAUGCUGACCUG
siCTL sense	UUGUAUGCAGUAUGUCCAUGC
siCTL anti-sense	AGAGACAUACUGCAUAUCAUG

Table S6. Sequences of the shRNAs used in the study.

Primer names	Sequences (5'-3')
shPCK2-1 sense	GGUUGGUCUCAAACUCCUAAC
shPCK2-1 anti-sense	UAGGAGUUUGAGACCAACCUG
shPCK2-2 sense	CGUUGAUUAAACAGUUAAACA
shPCK2-2 anti-sense	UUUAACUGUUUAAUCAACGUU
shPCK1-1 sense	GGUCGUGUUCGAAGUCCAAGG
shPCK1-1 anti-sense	UUGGACUUCGAACACGACCAG
shCTL sense	UUGUAUGCAGUAUGUCCAUGC
shCTL anti-sense	AGAGACAUACUGCAUAUCAUG

Table S7. List of antibodies used in this study.

Antigens	Manufacturer	Catalog Number	Application
NRF2	Proteintech	16396-1-AP	1:2000 for WB
SLC7A11	Proteintech	26864-1-AP	1:1000 for WB
GPX4	Proteintech	67763-1-Ig	1:3000 for WB, 1:200 for IHC
COX2	Proteintech	12375-1-AP	1:2000 for WB, 1:200 for IHC
ACSL4	Proteintech	81196-1-RR	1:3000 for WB, 1:200 for IHC
TFRC	Proteintech	10084-2-AP	1:3000 for WB
Pan-Kla	PTM Biolab	PTM-1401RM	1:500 for WB, 1:100 for IHC
PCK2	Proteintech	14892-1-AP	1:800 for WB, 1:200 for IHC, 1:100 for CoIP
$PCK2^{K100lac}$	PTM Biolab	-	1:500 for WB, 1:100 for IHC
KAT8	Abcam	ab200660	1:1000 for WB, 1:100 for CoIP
FTL	Proteintech	10727-1-AP	1:100 for IHC
OXSM	Proteintech	16642-1-AP	1:1000 for WB
OXSM	Abcam	ab154616	1:100 for CoIP
OGDH	Proteintech	82773-1-RR	1:2000 for WB
NDUFA6	Proteintech	15445-1-AP	1:2000 for WB
Parkin	Proteintech	14060-1-AP	1:3000 for WB, 1:100 for CoIP
MYC	Proteintech	60003-2-Ig	1:3000 for WB, 1:100 for CoIP
GST	Proteintech	66001-2-Ig	1:3000 for WB
Flag	Proteintech	66008-4-Ig	1:5000 for WB, 1:200 for CoIP
HA	Proteintech	51064-2-AP	1:3000 for WB, 1:200 for CoIP
His	Proteintech	66005-1-Ig	1:2500 for WB
IgG	CST	#3900	1:500 for CoIP
Lamin B1	Proteintech	66095-1-Ig	1:5000 for WB
β-actin	Proteintech	66009-1-Ig	1:2500 for WB

Table S8. Sequences of the primers used in the study.

Primer names	Sequences (5'-3')	Species
PCK2 forward	GCCATCATGCCGTAGCATC	*Homo sapiens*
PCK2 reverse	AGCCTCAGTTCCATCACAGAT	*Homo sapiens*
PCK2 forward	ATGGCTGCTATGTACCTCCC	*Mus musculus*
PCK2 reverse	GCGCCACAAAGTCTCGAAC	*Mus musculus*
FTH forward	CCCCCATTTGTGTGACTTCAT	*Homo sapiens*
FTH reverse	GCCCGAGGCTTAGCTTTCATT	*Homo sapiens*
FTL forward	CAGCCTGGTCAATTTGTACCT	*Homo sapiens*
FTL reverse	GCCAATTCGCGGAAGAAGTG	*Homo sapiens*
GPX4 forward	GAGGCAAGACCGAAGTAAACTAC	*Homo sapiens*
GPX4 reverse	CCGAACTGGTTACACGGGAA	*Homo sapiens*
COX2 forward	CTGGCGCTCAGCCATACAG	*Homo sapiens*
COX2 reverse	CGCACTTATACTGGTCAAATCCC	*Homo sapiens*
NDUFA6 forward	CGCCAAGCTACTTCTACCGC	*Homo sapiens*
NDUFA6 reverse	TCGGACTTTATCCCGTCCCA	*Homo sapiens*
NDUFB9 forward	GTGGTGCGTCCAGAGAGAC	*Homo sapiens*
NDUFB9 reverse	GGCCTTCGCCATATCCTTTTC	*Homo sapiens*
SDHAF1 forward	TGAGCCTGTACCGCGATCT	*Homo sapiens*
SDHAF1 reverse	GGTTCCTTGGACTGTCGCC	*Homo sapiens*
SDHAF3 forward	CCGGACCTCAAATCCCTGG	*Homo sapiens*
SDHAF3 reverse	CTCGTCAGAACCAACGGTCTT	*Homo sapiens*
LYRM7 forward	GGACGGGCAGTCAAGGTTTTA	*Homo sapiens*
LYRM7 reverse	GGTGCATCACAATATGGCACATT	*Homo sapiens*
FMC1 forward	TCCCCGTCGCACACTTTTC	*Homo sapiens*
FMC1 reverse	TGAAGCTCATGTTGGGCTCTG	*Homo sapiens*
OGDH forward	GGCTTCCCAGACTGTTAAGAC	*Homo sapiens*
OGDH reverse	GCAGAATAGCACCGAATCTGTTG	*Homo sapiens*
CS forward	TGCTTCCTCCACGAATTTGAAA	*Homo sapiens*
CS reverse	CCACCATACATCATGTCCACAG	*Homo sapiens*
IDH2 forward	CGCCACTATGCCGACAAAAG	*Homo sapiens*
IDH2 reverse	ACTGCCAGATAATACGGGTCA	*Homo sapiens*
MDH1 forward	GGTGCAGCCTTAGATAAATACGC	*Homo sapiens*
MDH1 reverse	AGTCAAGCAACTGAAGTTCTCC	*Homo sapiens*
MDH2 forward	TCGGCCCAGAACAATGCTAAA	*Homo sapiens*
MDH2 reverse	GCGGCTTTGGTCTCGATGT	*Homo sapiens*
NDUFA6 forward	TCGGTGAAGCCCATTTTCAGT	*Mus musculus*
NDUFA6 reverse	CTCGGACTTTATCCCGTCCTT	*Mus musculus*
NDUFB9 forward	AAGGTGCTGCGGCTGTATAAG	*Mus musculus*
NDUFB9 reverse	TCATCAAGCAAGCAAAGTACCG	*Mus musculus*
SDHAF1 forward	ATTTACCGATGGGACCCAGAC	*Mus musculus*
SDHAF1 reverse	GTCCGCACTTATTCAGATCCAC	*Mus musculus*
SDHAF3 forward	GGCGAATCTTGCTGCTACATC	*Mus musculus*
SDHAF3 reverse	GGACCAACAGTCTTATGTCTCCT	*Mus musculus*
LYRM7 forward	GTCAGCCCGCCAAGGTTTTA	*Mus musculus*
LYRM7 reverse	CAGTACGGCACATTTTCTGTGA	*Mus musculus*
FMC1 forward	CTTTCCGAGCACATCGGGTTA	*Mus musculus*
FMC1 reverse	CCGGATGCTACTCAAAAGGCA	*Mus musculus*
OGDH forward	GTTTCTTCAAACGTGGGGTTCT	*Mus musculus*
OGDH reverse	GCATGATTCCAGGGGTCTCAAA	*Mus musculus*

CS forward	GGACAATTTTCCAACCAATCTGC	*Mus musculus*
CS reverse	AGTCAATGGCTCCGATACTGC	*Mus musculus*
IDH2 forward	GGAGAAGCCGGTAGTGGAGAT	*Mus musculus*
IDH2 reverse	GGTCTGGTCACGGTTTGGAA	*Mus musculus*
MDH1 forward	GAACCAATCAGAGTCCTTGTGAC	*Mus musculus*
MDH1 reverse	GGCACAGTCTTGCAGTTCCA	*Mus musculus*
MDH2 forward	GCAACCCCTTTCACTCCTG	*Mus musculus*
MDH2 reverse	TCTGGTCTCAATGTGACTCAGAT	*Mus musculus*
β-actin forward	CATGTACGTTGCTATCCAGGC	*Homo sapiens*
β-actin reverse	CTCCTTAATGTCACGCACGAT	*Homo sapiens*
β-actin forward	GGCTGTATTCCCCTCCATCG	*Mus musculus*
β-actin reverse	CCAGTTGGTAACAATGCCATGT	*Mus musculus*
